# A database and framework for carbon ore resources and associated supply chain data

**DOI:** 10.1016/j.dib.2021.107761

**Published:** 2021-12-25

**Authors:** Devin Justman, Michael Sabbatino, Scott Montross, Scott Pantaleone, Andrew Bean, Kelly Rose, Randal B. Thomas

**Affiliations:** a National Energy Technology Laboratory, 1450 Queen Ave. SW, Albany, OR 97321, USA; b NETL Support Contractor, 1450 Queen Ave. SW, Albany, OR 97321, USA

**Keywords:** Carbon, Ore, Coal, Supply chain, Geochemistry, Geology, Infrastructure, Samples

## Abstract

The Carbon Ore Resources Database (CORD) is a working collection of 399 data files associated with carbon ore resources in the United States. The collection includes spatial/non-spatial, filtered, processed, and secondary data files with original data acquisition efforts focused on domestic coal resources. All data were acquired via open-source, online sources from a combination of 18 national, state, and university entities. Datasets are categorized to represent aspects of carbon ore resources, to include: Geochemistry, Geology, Infrastructure, and Samples. Geospatial datasets are summarized and analyzed by record and dataset density or the number of records or datasets per 400 square kilometer grid cells. Additionally, the “CORD Platform,” an ArcGIS Online geospatial dashboard web application, enables users to interact and query with CORD datasets. The CORD provides a single database and location for data-driven analytical needs associated with the utilization of carbon ore resources.

**Specifications Table**

**Table:** 

Subject	Energy Economics
Specific subject area	Carbon ore resources including in-situ coal resource geology and geochemistry, supply chains, waste streams, and beneficial uses.
Type of data	Tables Geodatabases Feature classes/shapefiles Rasters Figures
How data were acquired	All data were acquired from online databases using standard configuration PC and internet browser software.
Data format	Secondary Filtered Processed
Parameters for data collection	Data were collected for relevance to domestic US coal resources.
Description of data collection	The Carbon Ore Resources Database (CORD) is a working collection of spatial/non-spatial, filtered, processed, and secondary datasets categorized to represent aspects of US carbon and coal resources, including supply chains, waste streams, and end uses.
Data source location	Data were downloaded from various national, state, and university entities. Primary data sources: Alaska Division of Department of Geological & Geophysical Surveys (ADGGS) Arkansas Geological Survey (AGS) Environmental Integrity Project (EIP) Duke University/University of Kentucky (DU/UK) Illinois State Geological Survey (ISGS) Indiana Geological and Water Survey (IGWS) Kentucky Geological Survey (KGS) National Energy Technology Laboratory (NETL) Oklahoma Geological Survey (OGS) Pennsylvania Spatial Data Access (PASDA) Pennsylvania State University (PSU) SkyTruth Texas Railroad Commission (TRC) United States Department of Labor- Mine Safety and Health Administration (MSHA) United States Energy Information Administration (EIA) United States Geological Survey (USGS) West Coast Regional Carbon Sequestration Partnership (WESTCARB) Wyoming State Geological Survey (WSGS)
Data accessibility	Repository name: Energy Data eXchange (EDX) Data identification number: 10.18141/1,813,861 Direct URL to data: https://edx.netl.doe.gov/dataset/cord

**Value of the Data**

• The Carbon Ore Resources Database (CORD) enables broader understanding and data-driven analyses of in-situ-, supply chain-, and consumer- based carbon resources, by providing a single location to efficiently access carbon ore resource datasets for a range of applications and end users. The systematized database organizes carbon ore data so it can easily be retrieved and analyzed.
• Increased accessibility to systematized carbon ore resource datasets benefits research and development scientists, analysts, developers, economists, and engineers from various organizations. These entities include coal mining companies; power plant operators; government agencies; non-governmental organizations (NGOs); and natural resource managers.
• Access to integrated, comprehensive carbon ore resource data are necessary for a range of applications, including optimizing coal production and deliveries to existing and new markets; mitigating the impacts of coal ash disposal, acid mine drainage, and greenhouse gas emissions; increase beneficial use of coal and coal by-products; and extraction of specific coal sources for carbon-based products and rare earth elements.
• Broader applications include decision support for carbon management and policy, identifying opportunities for the development of coal and carbon management technologies.
• Geospatial datasets within the CORD facilitate mapping and analysis using GIS (Geographic Information Systems) software.

## 1 Data Description

The Carbon Ore Resources Database (CORD) is a collection of 399 individual data files associated with carbon ore resources. The original data acquisition efforts focused on coal resources in the United States. Supplementary File 1 provides descriptions for each individual data file organized by category. Supplementary File 2 lists each file by name, category, data type (secondary or processed), coal filter field (name of field used to filter coal related records), data format type (spatial (vector), spatial (raster), or table), available formats, source organization, link to the data download source, and publication citation (if available). The CORD can be downloaded from the NETL's Energy Data eXchange website (https://edx.netl.doe.gov/dataset/cord) as two separate zipped folders, one in a geodatabase format and the other in a folder file structure.

A summary of the number of data files and records within the CORD by category and data type or general format of the data are shown in Table 1. Data types include either tables or spatial formats. Table datasets include CSV (comma separated value) or Geodatabase table formats, while spatial data include feature classes within a Geodatabase or shapefiles (vector formats) and File Geodatabase Rasters (FGDBR) or TIFF (Tagged Image File Format) raster formats (Supplementary File 2). In total there are 20 table datasets containing 648,107 records, 238 vector datasets containing 745,232 records, and 141 individual raster datasets (Table 1). Most of these data files (87% of total) and records (79% of total) are contained within the Geology category (Table 1). The data are organized into 6 categories (Table 1): Geochemistry, Geology, Infrastructure, Infrastructure network, Samples integrated, and Samples original. These categories are described in further detail below.

**Table 1:** Number of data files and records by data type (general format) and category contained within the CORD.

Category	Data format type	File count	Record count
Geochemistry	Spatial (vector)	6	3928
	Table	1	84
Geochemistry Total		7	4012

Geology	Spatial (raster)	141	141
	Spatial (vector)	203	526,806
	Table	3	547,118
Geology Total		347	1074,065

Infrastructure	Spatial (vector)	16	88,418
	Table	1	35,359
Infrastructure Total		17	123,777

Infrastructure network (Total)	Spatial (vector)	9	90,634

Samples integrated	Spatial (vector)	1	28,560
	Table	1	36,216
Samples integrated Total		2	64,776

Samples original	Spatial (vector)	3	6886
	Table	14	29,330
Samples original Total		17	36,216

Grand Total		399	1393,480

*Note:* Individual raster data files are counted as a single record.

The number of data files and records within the CORD by primary source organization and data type or general format of the data is shown in Table 2. In total, there are 397 data files containing 1328,704 records from 18 primary sources (organizations). Most of the data files (74% of total) and records (72% of total) are sourced from the USGS [1], [2], [3], [4], [5], [6], [7], [8], [9], [10], [11], [12].

**Table 2:** Number of data files and records by data type (general format) and primary source organization.

Primary source organization	Data type	File count	Record count
Alaska Division of Department of Geological & Geophysical Surveys (ADGGS)	Spatial (vector)	5	434
	Table	2	22
Arkansas Geological Survey (AGS)	Table	1	6
Duke University/University of Kentucky (DU/UK)	Table	1	171
Environmental Integrity Project (EIP)	Spatial (vector)	1	737
Illinois State Geological Survey (ISGS)	Spatial (vector)	69	135,222
	Table	1	5642
Indiana Geological and Water Survey (IGWS)	Spatial (vector)	1	3125
Kentucky Geological Survey (KGS)	Table	1	3238
National Energy Technology Laboratory (NETL)	Table	1	1028
Oklahoma Geological Survey (OGS)	Table	3	31,576
Pennsylvania Spatial Data Access (PASDA)	Spatial (vector)	1	13,405
Pennsylvania State University (PSU)	Table	1	1468
SkyTruth	Spatial (vector)	1	48,529
Texas Railroad Commission (TRC)	Spatial (vector)	2	722
United States Department of Labor- Mine Safety and Health Administration (MSHA)	Spatial (vector)	1	1720
	Table	1	35,359
United States Energy Information Administration (EIA)	Spatial (vector)	7	88,805
United States Geological Survey (USGS)	Spatial (raster)	141	141
	Spatial (vector)	147	423,900
	Table	7	533,381
West Coast Regional Carbon Sequestration Partnership (WESTCARB)	Spatial (vector)	1	66
Wyoming State Geological Survey (WSGS)	Spatial (vector)	1	7
Grand Total		397	1328,704

*Note:* Individual raster data files are counted as a single record.

**Fig. 1:**
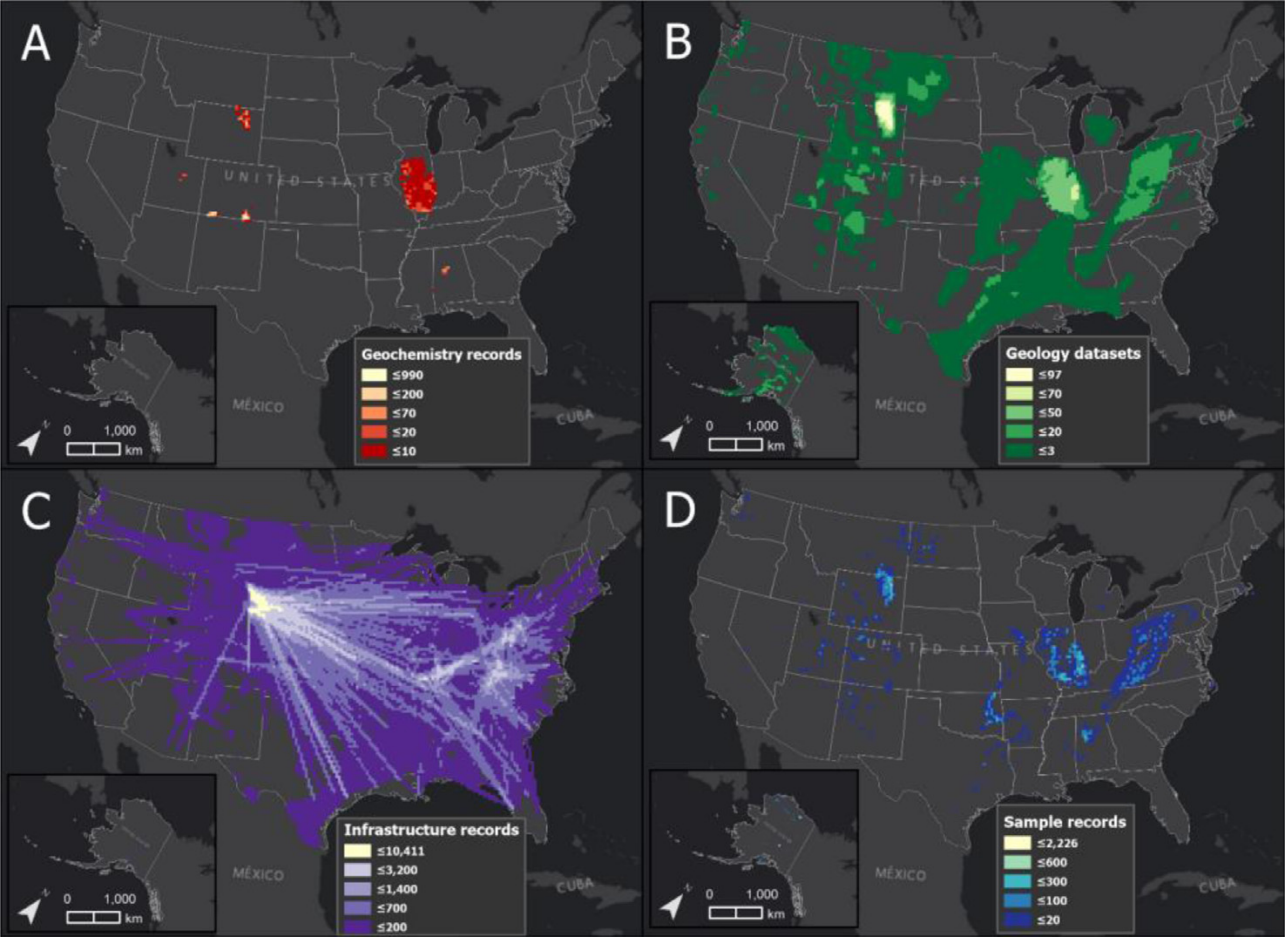
displays spatial data summarization of database categories within 400 sq. km grid cells across the Unites States, including Alaska. Fig. 1A (top left) displays the quantity of spatial records contained within the “Geochemistry” category. Fig. 1B (top right) displays the quantity of spatial datasets contained within the “Geology” category. Fig. 1C (bottom left) displays the quantity of spatial records contained within the “Infrastructure” and “Infrastructure network” categories. Fig. 1D (bottom right) displays the quantity of spatial records contained within the “Samples integrated” category.

The “Geochemistry” category consists of seven secondary data files and 4012 records associated with coal geochemistry but not explicitly coal sample data (Table 1). This includes data that are derivatives of or associated with coal sample geochemistry, for example, interpolations of elemental concentrations or water produced from coal beds. These data are sourced from the USGS [1,2] and ISGS [13] (Table 2). Fig. 1A displays the quantity of spatial records within the “Geochemistry” category across the United States and Alaska within 400 sq. km grid cells.

The “Geology” category consists of 347 secondary data files and 1,074,065 records associated with coal geology (Table 1). This includes stratigraphic records from wellbores, coal fields, coal basins, and interpretation of coal bed/seam geometry (i.e., thickness, surface elevation, overburden). These data are sourced from the USGS [3], [4], [5], ISGS [13], ADGGS [14], OGS [15], and WESTCARB [16] (Table 2). Fig. 1B displays the quantity of spatial datasets within the “Geology” category across the United States and Alaska within 400 sq. km grid cells.

The “Infrastructure” category consists of 17 secondary data files and 123,777 records associated with coal resource infrastructure (Table 1). Currently, this includes datasets related to coal mines and power plants. These data are sourced from the USGS [6], ISGS [13], EIP [17], MSHA [18], PASDA [19], SkyTruth [20], and the TRC [21] (Table 2). Fig. 1C displays the quantity of spatial records within the “Infrastructure” and “Infrastructure network” categories across the United States and Alaska within 400 sq. km grid cells.

**Fig. 2:**
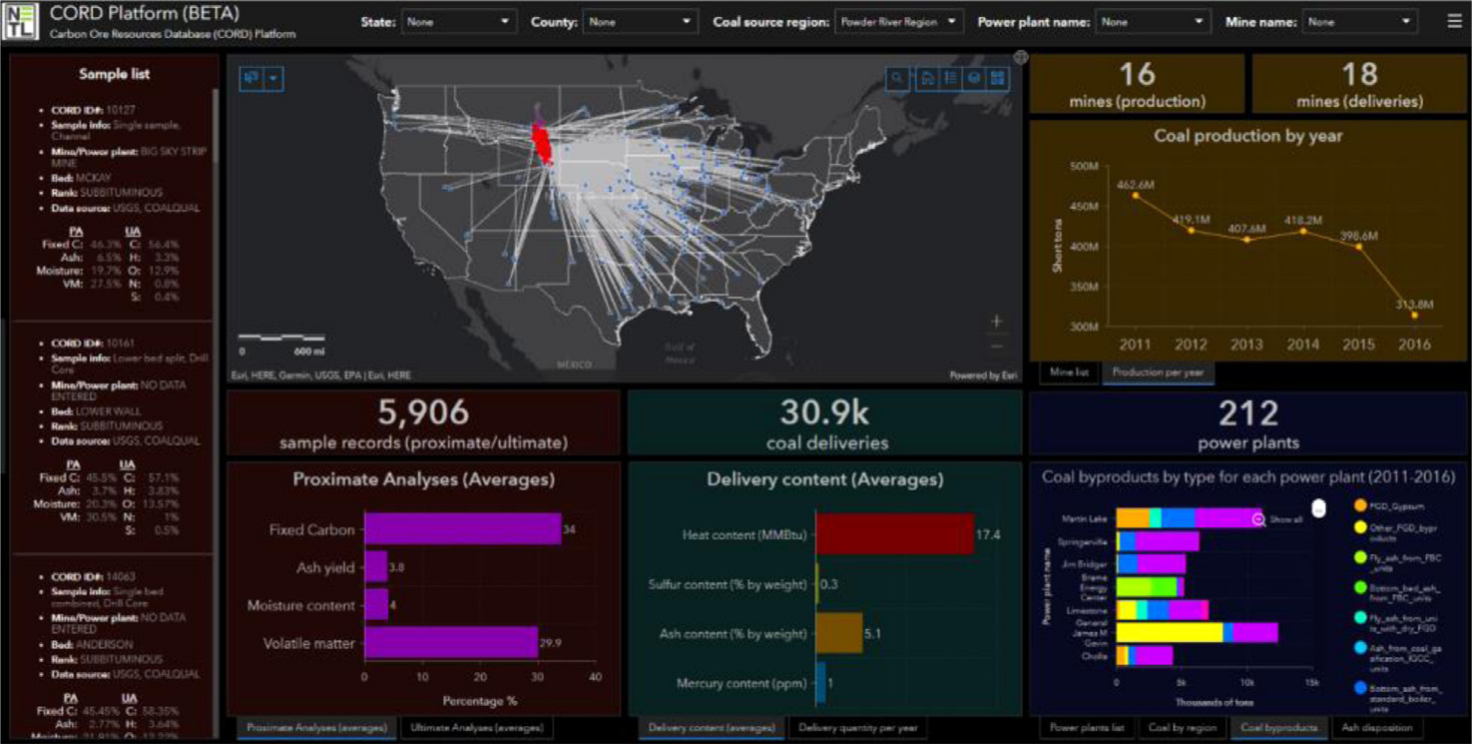
displays an example and screenshot of the “Infrastructure network” datasets used within a dashboard web application entitled, “CORD Platform”. The map (top center) displays coal delivery pathways extending from mine (black squares) to power plants (blue triangles) and coal samples (red circles) associated with the Powder River (Basin) coal source region. Datasets are summarized within charts, lists, and graphs accordingly.

The “Infrastructure network” category consists of nine processed data files and 90,634 records associated with coal resource infrastructure (Table 1). Currently, this includes datasets related to coal supply chains from mines to power plants (i.e., sources, production, deliveries, consumption, stockpiles, by-products) from 2011 through 2016. These data are sourced from the USGS [3] MSHA [18], and the EIA [22], [23], [24] (Table 2). Fig. 1C displays the quantity of spatial records within the “Infrastructure” and “Infrastructure network” categories across the United States and Alaska within 400 sq. km grid cells. Additionally, Fig. 2 displays these datasets used within an online dashboard web application, entitled “CORD Platform”. Link to the application is provided through EDX (https://edx.netl.doe.gov/dataset/cord)

The “Samples integrated” category consists of two processed data files and 64,776 records associated with coal samples (Table 1). This includes datasets integrated from the “Samples original” category. The two datasets are both the integration of all collected coal sample records. One is provided in a table (“Samples_All”) and the other in a spatial format (“Samples_spatial”). Fig. 1D displays the quantity of spatial records within the “Samples_spatial” dataset within the “Samples integrated” category across the United States and Alaska within 400 sq. km grid cells.

The “Samples original” category consists of 17 processed and secondary data files and 36,216 records associated with coal samples (Table 1). This includes various types of geochemical analyses associated with coal samples (i.e., metadata, physical properties, proximate/ultimate, oxides, trace elements, etc.). These data are sourced from the USGS [7], [8], [9], [10], [11], [12] OGS [15], ADGGS [25,26], AGS [27], DU/UK [28], ISGS [29], IGWS [30], KGS [31], NETL [32], PSU [33], and the WSGS [34] (Table 2). Fig. 1D displays the quantity of spatial records within the “Samples_spatial” dataset within the “Samples integrated” category (same as “Samples integrated” category) across the United States and Alaska within 400 sq. km grid cells.

A data dictionary (field names and descriptions) is provided in an Excel workbook for datasets that required additional processing and integration steps (Supplementary File 3), where each tab refers the following 10 datasets:

• **Samples_All** (tab “Samples_All”)
• **Coal_Delivery_Pathways_2011_2016** (tab “Coal_Del_Pathways_2011_2016”)
• **Coal_Mine_Deliveries_2011_2016** (tab “Coal_Mine_Del_2011_2016”)
• **Coal_Mine_Production_2011_2016** (tab “Coal_Mine_Prod_2011_2016”)
• **Coal_Source_Regions_Production_Deliveries_2011_2016** (tab “Coal_Source_Regions_2011_2016”)
• **Power_Plant_ByProductsType_2011_2016** (tab “PowerPlant_ByProdType_2011_2016”)
• **Power_Plant_Consumption_2011_2016** (tab “PowerPlant_Cons_2011_2016”)
• **Power_Plant_Deliveries_and_ByProducts_2011_2016** (tab “PowerPlant_Del_ByProd_2011_2016”)
• **Power_Plant_Deliveries_by_Coal_Source_Region_2011_2016** (tab “PowerPlant_Del_by_CSR_2011_2016”)
• **Power_Plant_Stockpiles_2011_2016** (tab “PowerPlant_Stock_2011_2016”)

Additionally, two python scripts and two CSV files associated with field mapping of the “Samples_All” table are provided in Supplementary File 4:

• **CORD_Data_Script1.py** - This script takes an input folder path with CSV files and exports the files into a new folder with updated and modified attribute names.
• **CORD_Data_Script2.py** - This script takes an input folder path with CSV files and exports the combined files into a new folder with an updated schema and all empty rows removed.
• **CORD_Field_map.csv** - This CSV file provides the Mapping of the input data fields to the output data fields for the data conversion script CORD_Data_Script1.py.
• **CORD_Schema_combined.csv** - This CSV file contains the combined schema for the data that is converted from multiple input CSV files to a single output CSV. It is used with the CORD_Data_Script2.py python script.

## 2 Experimental Design, Materials and Methods

All data processing was performed using ESRI's ArcGIS ArcMap 10.7 software [35], Microsoft Excel, and python scripting. The data collection method consisted of manual internet searches through authoritative sources starting at the national level and then at the state level. Searches were focused on explicit coal datasets and data that do not primarily include coal information (i.e., the USGS produced water database [1]). Data were also collected from journal publications where readily available (i.e., Taggart et al. [28]). As these data were collected, they were catalogued and categorized into file folders according to their category (Table 1) and converted to a table (File Geodatabase Table or CSV) or spatial file (feature class, shapefile, FGDBR, or TIFF) format. Where applicable, each data file was renamed according to location (state or geologic basin), name and/or physical representation, and source organization acronym (i.e., AppBasin_Pocahontas_Coal_Bed_Thickness_USGS). To summarize overlapping datasets and features (records) by category within 400 sq. km grid cells (Fig. 1), the Cumulative Spatial Impact Layers (CSIL) tool [36] was applied within ArcMap 10.7 software [35].

Secondary data files that did not require any processing were directly imported into the database. If data required filtering for explicit coal records, the field name used to filter the records was recorded Supplementary File 2, within the “Coal_filter_field”. Files labelled as “processed” in Supplementary File 2, required additional modification before including into the database. These data files include those in the “Infrastructure network”, “Samples original”, and “Samples integrated” categories. Each processed data file involves a unique method before integrating into the CORD. These methods are described by category and for each processed data file as necessary:

Infrastructure network:

**Coal_Source_Regions_Production_Deliveries_2011_2016**: This dataset was created from the original secondary data file “Coal_fields_USGS” [3]. The 602 polygons representing coal fields and basins were dissolved by name (i.e., areas with the same name were merged into a single record.) and then manually split in key areas. The Appalachian Basin Region was split into North, Central, and Southern regions by county boundaries as in the coal basins map within the EIA Coal Data Browser (https://www.eia.gov/coal/data/browser/). This North Appalachian Basin Region was further split to separate the Pennsylvania Anthracite Region. In total, 109 separate coal source regions were developed. Coal production and delivery quantities information from 2011 through 2016 were added by spatially joining (point features closest to each region) and summing values from the “Coal_Mine_Production_2011_2016” and “Coal_Mine_Deliveries_2011_2016”, respectively.

**Coal_Delivery_Pathways_2011_2016:** This dataset was extracted from the “Page 5 Fuel Receipts and Costs” tab in the EIA-923 excel files [22] for the years 2011 through 2016. Annual spreadsheets were compiled into one CSV file, filtered for “Coal” in the “Fuel Group” field, and exports to other countries were removed, using the “Plant State” field. The table was then pivoted on delivery quantity or “QUANTITY” field to obtain new field totals by month and year(s). A field was added to designate interstate or intrastate (“InterIntraState”) deliveries. To obtain latitude and longitude coordinates, mine coordinate information was joined from the MSHA mines dataset using the unique MSHA unique identifier fields. Before joining, the raw mine longitude coordinates first had to be multiplied by −1, to correctly represent the decimal degrees format. Additionally, locations with null or visibly incorrect coordinates were corrected by comparing the location description (“DIRECTIONS” and “NEAREST_TO” fields) with satellite imagery in Google Maps. A total of 14 mine locations associated with deliveries (2011–2016) were corrected. This processing procedure for mine location correction was repeated for “Coal_Mine_Production_2011_2016” dataset as well. For delivery records that did not have a unique MSHA identifier number, the centroid coordinates of the associated counties (“COALMINE_COUNTY”) or states (“COALMINE_STATE”; if county information is unavailable) were used. These centroids were obtained by calculating the centroids in county and state polygons in the “WGS84” datum. A total of 132 delivery records could not be mapped due to lack of spatial information and were left out. To obtain latitude and longitude coordinates for power plants, the original data were extracted from “Plant” tab in the annual EIA-860 excel files (“2__Plant_Y[YEAR].xlsx”) for the years 2012 (locations not available before 2012) through 2016 [23]. Annual spreadsheets were combined into one CSV file and pivoted on the unique identifying field for power plants (“Plant Code”) to obtain a single unique record for each location. Power plant coordinates were then joined to the coal deliveries table using the “Plant Code” field. Polylines or delivery origin-destination paths from mines to power plants were created using the “XY to Line” tool within ArcMap 10.7 software [35]. After the spatial data was created, polyline lengths were calculated in kilometers using the North America Lambert Conformal Conic projection.

**Coal_Mine_Deliveries_2011_2016:** This dataset was created from the “Coal_Delivery_Pathways_2011_2016” dataset, by dissolving on the MSHA unique identifying number, latitude, and longitude fields to obtain a single unique record for each mine. Delivery quantities were aggregated and summed for each and all years, including total delivery count from each mine. Point features were then created to represent individual mines. To obtain the name of the coal source region associated with each mine (“Coal_Source_Region”), the “Coal_Source_Region_2011_2016” dataset was spatially joined to the mine point features (by nearest coal source region polygon). The mine point features were then joined to the “Coal_Delivery_Pathways_2011_2016” dataset” by a temporary “Delivery_ID” field (deleted after join), to obtain the coal source region names within the deliveries dataset.

**Coal_Mine_Production_2011_2016:** This was extracted from the EIA-7A excel files [24] for the years 2011 through 2016 and compiled into one CSV file. The table was then pivoted on the “YEAR” field to obtain a unique record each mine and new field totals for production quantities were summed for each and all year(s) (“p_[YEAR]”). Mine latitude, longitude, and metadata fields were added by joining the MSHA unique identifying numbers within the “MINE_ID” field within the MSHA mines dataset [18]. Although latitude and longitude were available in the EIA-7A data [24], the MSHA mines dataset [18] was used due to inconsistent location and name values from year to year. Using the same mine relocation method used for the “Coal_Delivery_Pathways_2011_2016” dataset, a total of 23 mine locations were corrected. Point features were then created to represent individual mines. To obtain the name of the coal source region associated with each mine (“Coal_Source_Region”), the “Coal_Source_Region_2011_2016” dataset was spatially joined to the mine point features (by nearest coal source region polygon).

**Power_Plant_Deliveries_by_Coal_Source_Region_2011_2016:** This dataset was extracted from the “Coal_Delivery_Pathways_2011_2016”dataset. First, the “Coal_Delivery_Pathways_2011_2016” dataset was dissolved on the unique identifying number for power plants (“Plant_code”) and “Coal_Source_Region” fields to obtain a single unique record for each unique combination of power plant and coal source region. Delivery quantities were aggregated and summed for each and all years. The “Coal_Source_Region” field was then pivoted to add fields for coal delivery quantity totals for each unique combination of region and year. Point features were then created to represent individual power plants.

**Power_Plant_Deliveries_and_ByProducts_2011_2016:** This dataset was extracted from the “Coal_Delivery_Pathways_2011_2016” dataset and the “8A Annual Byproduct Disposition” tab in the EIA-923 excel files [22] for the years 2011 through 2016. First, the “Coal_Delivery_Pathways_2011_2016” dataset was dissolved on the unique identifying number for power plants (“Plant_code”), latitude, and longitude fields to obtain a single unique record for each power plant. Delivery quantities were aggregated and summed for each and all years (total average as well). Point features were then created to represent individual mines. Next, the “8A Annual Byproduct Disposition” annual spreadsheets [22] were compiled into one CSV file, and then pivoted on “Year” and “Plant ID” fields to obtain by-product quantity totals for each combination of disposition type (sold, stored, used, disposed) and year for each power plant. The point features containing the delivery quantity information were then joined to the by-product information using the unique identifying “Plant code” and “Plant ID” fields.

**Power_Plant_Consumption_2011_2016:** This dataset was extracted from the “Page 1 Generation and Fuel Data” tab within the EIA-923 excel files [22] for the years 2011 through 2016. Annual spreadsheets for consumption were compiled into a single CSV file and filtered on the “Physical Unit Label” field for values containing the term “short tons” to ensure only coal records were kept. The consumption information spreadsheet was then pivoted on “Year”, “Plant ID”, and “Reported Fuel Type Code” fields to create new fields for coal consumption quantity totals and the annual average coal heat content (calculated from monthly estimates) by coal rank (i.e., lignite, subbituminous, anthracite, etc.) and each and all years. Additionally, a field for the range in average heat content for all years (2011–2016) was added. Latitude and longitude coordinates, as well as plant name, state, and utility company name were joined using the “Plant Code” field in the compiled data from the annual EIA-860 excel files [23] (“2__Plant_Y[YEAR].xlsx”), described above in the “Coal_Delivery_Pathways_2011_2016” dataset. Point features were then created to represent individual power plants.

**Power_Plant_Stockpiles_2011_2016:** This dataset was extracted from the “Page 2 Coal Stocks Data” tab within the EIA-923 excel files [22] for the years 2011 through 2016. Annual spreadsheets for coal stockpiles were compiled into a single CSV and pivoted on “Year”, “Plant ID”, and “Reported Fuel Type Code” fields to create new fields for average annual coal stockpile quantities (calculated from monthly estimates) by coal rank (i.e., lignite, subbituminous, anthracite, etc.) and each and all years. Latitude and longitude coordinates, as well as plant name, state, and utility company name were joined using the “Plant Code” field in the compiled data from the annual EIA-860 excel files (“2__Plant_Y[YEAR].xlsx”) [23], described above in the “Coal_Delivery_Pathways_2011_2016” dataset. Point features were then created to represent individual power plants.

**Power_Plant_ByProductsType_2011_2016:** This dataset was extracted from the “8A Annual Byproduct Disposition” tab in the EIA-923 excel files [22] for the years 2011 through 2016. Annual spreadsheets were compiled into one CSV file, and then pivoted on “Year”, “Plant ID”, and “By Product Description” fields to obtain by-product quantity totals for each combination of disposition type (sold, stored, used, disposed), by-product type, and year for each power plant. Latitude and longitude coordinates, as well as plant name, state, and utility company name were joined using the “Plant Code” field in the compiled data from the annual EIA-860 excel files (“2__Plant_Y[YEAR].xlsx”) [23], described above in the “Coal_Delivery_Pathways_2011_2016” dataset. Point features were then created to represent individual power plants. Additional fields were created to sum coal ash by-product quantity totals for 2011 through 2016 by each disposition type (“Ash_[*disposition type*]_2011_2016”) and overall total (“Ash_Total_2011_2016”).

Samples original:

**AK_Coal_samples_AGDB_USGS:** The original data were extracted from the “Main”, “Chemistry”, and “Parameters” tables within the Alaska Geochemical Database (geodatabase format) [7]. The “Main” table was first filtered for the term “coal” within the “spec_name” field to filter and extract coal samples records, resulting in 839 unique records. Fields that had all null values were removed. The “Parameters” table was first joined to the “Chemistry” table using the “p” and “field_name” fields. This join table was then pivoted on the “element”, “units”, and “v” fields to create new fields for each unique combination of element type and their associated units with analytical values for each unique sample and analysis. A new “Sample_ID” fields was created to track the unique sample record and analysis type. This field was created by combining the text from the “lab_id” and “method” fields with an underscore (“_”). The filtered “Main” table was then joined to the table containing the geochemical analytical data using the “lab_id” field. The “analytic_method_desc”, “digestion_method”, and “pub id” fields from the “Methods” table were joined to the geochemical analytical data using the “analytic_method” and “method” fields, respectively. The “pub_id” field was then used to join the “url” and “notes” field from the “Bibliography” table.

**AK_Holitina_Basin_Coal_samples_ADGGS:** The original data were filtered for the term “coal” from the “ri2015–3-rock-eval-toc” CSV file [25]. The table was also converted into a file geodatabase table.

**AK_Jarvis_creek_coal_samples_ADGGS:** The original data were extracted from the “pir2018–2-jarvis-creek-coal-proximate-analysis”, “pir2018–2-jarvis-creek-coal-ultimate-analysis”, and “pir2018–2-jarvis-creek-coal-rock-eval-ro-toc” CSV files [26]. All CSV files were joined on the “sample_id” fields. Duplicate fields were removed, and the final table was converted to a file geodatabase table.

**AR_Coal_samples_AGS:** The original data were copied from “Table 1. Average Analysis of Arkansas Coals (as-received basis)” located directly on the AGS website [27], https://www.geology.arkansas.gov/energy/coal-in-arkansas.html under the “Chemical Analysis” heading. The entire table was pasted into an excel spreadsheet, converted to a CSV and file geodatabase table.

**Coal_Ash_samples_NETL:** The original data were extracted from the “Results (whole Sample Conc.)” and “Results (Ash Based Conc.)” tables or tabs within the “collected-samples-spreadsheet-v051515” excel file [32]. A new field, “Sample_type” was added to each table to define whether each record is a “Whole sample” or “Ash” sample. Both tables were then combined into one table as a CSV file and finally converted into a file geodatabase table.

**Coal_CCP_samples_USGS:** The original data [8] was directly converted from the “AllData” CSV file to a file geodatabase table.

**Coal_samples_NaCQI_USGS:** The original data were extracted from the “Descriptive data”, “Oxide analyses”, “Whole Coal – Remnant moisture”, “Whole Coal – Dry basis”, and “Proximate-Ultimate analyses” tables or tabs within the “NaCQI” excel file [9]. All tables were modified to ensure all fields were concatenated into one cell, moved into one (top) row, and empty rows were removed. All tables were then joined on the “Laboratory number” field, in one CSV file and duplicate fields were removed. The CSV file was converted into a file geodatabase table.

**Coal_samples_NGDB_USGS:** The original data were extracted from the “GEODATA”, “NAA”, “OTHER”, and “UNKNOWN” dbf files from the National Geochemical Database for Rocks [10]. The “GEODATA” dbf file was filtered using the term “coal” within the “SPEC_NAME” field. The “NAA” and “OTHER” dbf files were joined to the “GEODATA” dbf file using the “LAB_ID” field. Duplicate fields were removed. The resulting dbf file was converted into a CSV file and file geodatabase table.

**Coal_samples_PSU_Energy_Institute_Coal_Bank:** The original data were downloaded from the PSU Energy Institute Coal Sample Bank (http://www.energy.psu.edu/copal/index.html) [33] query form. All available data were compiled into a CSV file and converted into a file geodatabase table.

**COALQUAL_USGS:** The original data were extracted from the “COALQUAL” point feature class, “Oxide”, “Proximate_Ultimate”, and “Trace_Elements” and file geodatabase tables within the “COALQUAL” geodatabase [11]. All file geodatabase tables were joined to the “COALQUAL” point feature class using the “Sample_ID” or “SampleNo” fields. Duplicate fields were deleted, and the joined table was saved as a file geodatabase table and CSV file.

**Fly_Ash_Samples_Taggart:** The original data were downloaded from the supplementary data associated with the Taggart et al. (2016) journal publication [28] from DU/KU. The data were extracted from the “HNO3-extractable” tab within the “es6b00085_si_002” excel file and copied into a CSV file. Fields were modified to ensure they were in a single top row. A “Sample_type” field was added with all values set to “Fly ash”. This CSV file was then converted to a file geodatabase table.

**IL_Coal_quality_samples_ISGS:** The original data was directly converted from the “coal-quality-nonconf” excel file [29] to a CSV file and file geodatabase table.

**IN_Coal_quality_samples_DB_IGWS:** The dataset [30] did not require processing but was additionally convert to a CSV file.

**KY_Coal_quality_samples_KGS:** The original data were extracted from the “borehole_12,182,019_19,372”, “physicalPropsAnaly”, “proximateAnaly”, “ultimateAnaly”, “wholeTraceAnaly”, and “ashTraceAnaly” excel files from the KGS. All but the 2 “TraceAnaly” tables were joined using the “sample_number” field and saved out into a single CSV file [31]. The 2 “TraceAnaly” tables were merged into a single CSV file and a field was added (“ash_yes_no”) to define whether the trace element analytics were performed on an “ash” sample or not. The two resulting CSV files were combined with a full outer join using the “sample_number” fields. The resulting CSV file was then converted into a file geodatabase table. Duplicate fields were removed.

**OK_Coal_quality_samples_OGS:** The original data were extracted from the “Data-Coal-Analytical-Header” and “Data-Coal-Analytical-Data” excel files from the OGS [15]. Tables were joined on the “Point_ID” field and saved out into a CSV file. Duplicate fields were removed. Additionally, a “Sample_ID” fields was added, a concatenation of the “Point_ID” and “ID_Sub2” fields. The resulting CSV file was converted into a file geodatabase table.

**PRB_Trace_elements_Stratigraphy_USGS:** The original data was directly converted from the “waptg” shapefile [12] to a CSV file and file geodatabase table.

**WY_Coal_samples_WSGS:** The original data was extracted from the “Appendix 1”, “Appendix 2”, “Appendix 3” tabs within the “ri-71-ap” excel file from the WSGS [34]. All records from the 3 tables were combined into one CSV file, with all fields moved into a single top row. Explicit coal samples were filtered using the term “coal” within the “Deposit_type_category” field. The resulting CSV file was converted into a file geodatabase table.

Samples integrated:

**Samples_All:** This working dataset was created by manually mapping the fields within the “Samples original” datasets (CSV files) to a new single table schema (Supplementary File 4) in Excel. With the field mapping complete, the new table (CSV file) was populated with the new schema using several python scripts (Supplementary File 4). The resulting CSV file was converted into a file geodatabase table. Additionally, fields with all null values were deleted from the table.

**Samples_spatial:** This working dataset was created by converting the available latitude and longitude coordinates from the “Samples_All” dataset to a point feature class (spatial dataset).

## Ethics Statement

The authors declare that the work described is original and has not been submitted elsewhere for publication. No conflict of interest exists in this submission.

## CRediT authorship contribution statement

**Devin Justman:** Methodology, Software, Investigation, Data curation, Writing – original draft, Visualization. **Michael Sabbatino:** Software, Data curation, Methodology, Writing – review & editing. **Scott Montross:** Conceptualization, Writing – review & editing. **Scott Pantaleone:** Writing – review & editing. **Andrew Bean:** Writing – review & editing. **Kelly Rose:** Conceptualization, Supervision, Project administration, Funding acquisition. **Randal B. Thomas:** Conceptualization, Supervision, Writing – review & editing.

## Declaration of Competing Interest

The authors declare that they have no known competing financial interests or personal relationships which have or could be perceived to have influenced the work reported in this article.

## Appendix Supplementary materials

Image, application 1

Image, application 2

Image, application 3

Image, application 4

Image, application 5

Image, application 6

Image, application 7
