# Supplementary material for: A database and framework for carbon ore resources and associated supply chain data
Source: Data Brief. 2021 Dec 25;40:107761. doi: 10.1016/j.dib.2021.107761 (PMC8718727; doi:10.1016/j.dib.2021.107761)
Supplement: Supplementary file 1 [file mmc1.docx]

Supplementary File 1: Descriptions of individual data files within the Carbon Ore Resources Database (CORD) by category.

**Geochemistry (excluding sample data):**

- **IL_Herrin_Chlorine_Py_ISGS:** A (vector) spatial dataset consisting of 150 polygon areas or records representing Chlorine content (percent) of the Herrin coal bed in the state of Illinois.
- **IL_Herrin_RankBtu_Py_ISGS:** A (vector) spatial dataset consisting of 92 polygon areas or records representing heat content (million BTU/short ton) of the Herrin coal bed in the state of Illinois.
- **IL_Herrin_Sulfur_Py_ISGS:** A (vector) spatial dataset consisting of 128 polygon areas or records representing Sulfur content (pounds/million BTU) of the Herrin coal bed in the state of Illinois.
- **IL_Springfield_RankBtu_Py_ISGS:** A (vector) spatial dataset consisting of 48 polygon areas or records representing heat content (in million Btu/short ton) of the Springfield coal bed in the state of Illinois.
- **IL_Springfield_Sulfur_Py_ISGS:** A (vector) spatial dataset consisting of 60 polygon areas or records representing Sulfur content (pounds/million BTU) of the Springfield coal bed in the state of Illinois.
- **Western_US_Basins_vitrinite_reflectance_USGS:** A table consisting of 84 records representing vitrinite reflectance (percent) variations in coal core samples of Paleocene-Eocene aged coals of the Powder River, Williston, Hanna, Bighorn, and Bull Mountain basins.
- **Produced_waters_coal_geochem_USGS:** A (vector) spatial dataset consisting of 3,450 point locations or records representing produced water geochemistry (from oil and gas wells) explicitly filtered for coal geology.

**Geology:**

- **AK_Coal_basins_ADGGS:** A (vector) spatial dataset consisting of 10 polygon areas or records representing boundaries of coal basins in Alaska.
- **AK_Coal_districts_contacts_ADGGS:** A (vector) spatial dataset consisting of 52 polygon areas or records representing coal districts or contacts showing limits of geologic units in Alaska.
- **AK_Coal_fields_ADGGS:** A (vector) spatial dataset consisting of 38 polygon areas or records representing boundaries of coal fields in Alaska.
- **AK_Coal_fields_WESTCARB:** A (vector) spatial dataset consisting of 66 polygon areas or records representing boundaries of coal fields in Alaska.
- **AK_Coal_occurrences_ADGGS:** A (vector) spatial dataset consisting of 150 point locations or records representing coal occurrences of unknown extent in Alaska.
- **AK_Coal_rank_boundaries_ADGGS:** A (vector) spatial dataset consisting of 184 polygon areas or records representing boundaries of coal rankings in Alaska.
- **AppBasin_Fireclay_Coal_Zone_Elevation_USGS:** A (vector) spatial dataset consisting of 3,793 polygon areas or records representing the boundary and elevation above sea level (feet) of the top of the Fireclay Coal Zone in the Appalachian Basin.
- **AppBasin_Fireclay_Coal_Zone_Overburden_USGS:** A (vector) spatial dataset consisting of 4,935 polygon areas or records representing the boundary and overburden (feet) of the Fireclay Coal Zone in the Appalachian Basin.
- **AppBasin_Fireclay_Coal_Zone_Thckness_USGS:** A (vector) spatial dataset consisting of 3,268 polygon areas or records representing the boundary and thickness (feet) of the Fireclay Coal Zone in the Appalachian Basin.
- **AppBasin_Lower_Kittanning_Coal_Zone_mined_out_USGS:** A (vector) spatial dataset consisting of 4,233 polygon areas or records representing the boundaries of mined out areas of the Lower Kittanning Coal Zone in the Appalachian Basin.
- **AppBasin_Lower_Kittanning_Coal_Zone_Outcrop_USGS:** A (vector) spatial dataset consisting of 2,237 polygon areas or records representing the outcrop boundaries of the Lower Kittanning Coal Zone in the Appalachian Basin.
- **AppBasin_Pittsburgh_Coal_Bed_Elevation_USGS:** A (vector) spatial dataset consisting of 4,776 polygon areas or records representing the boundary and elevation above sea level (feet) of the top of the Pittsburgh Coal Bed in the Appalachian Basin.
- **AppBasin_Pittsburgh_Coal_Bed_Overburden_USGS:** A (vector) spatial dataset consisting of 2,331 polygon areas or records representing the boundary and overburden (feet) of the Pittsburgh Coal Bed in the Appalachian Basin.
- **AppBasin_Pittsburgh_Coal_Bed_Thickness_USGS:** A (vector) spatial dataset consisting of 4,037 polygon areas or records representing the boundary and thickness (feet) of the Pittsburgh Coal Bed in the Appalachian Basin.
- **AppBasin_Pocahontas_Coal_Bed_Elevation_USGS:** A (vector) spatial dataset consisting of 333 polygon areas or records representing the boundary and elevation above sea level (feet) of the top of the Pocahontas Coal Bed in the Appalachian Basin.
- **AppBasin_Pocahontas_Coal_Bed_Overburden_USGS:** A (vector) spatial dataset consisting of 1,513 polygon areas or records representing the boundary and overburden (feet) of the Pocahontas Coal Bed in the Appalachian Basin.
- **AppBasin_Pocahontas_Coal_Bed_Thickness_USGS:** A (vector) spatial dataset consisting of 458 polygon areas or records representing the boundary and thickness (feet) of the Pocahontas Coal Bed in the Appalachian Basin.
- **AppBasin_Pond_Creek_Coal_Zone_Elevation_USGS:** A (vector) spatial dataset consisting of 3,255 polygon areas or records representing the boundary and elevation above sea level (feet) of the top of the Pond Creek Coal Zone in the Appalachian Basin.
- **AppBasin_Pond_Creek_Coal_Zone_Overburden_USGS:** A (vector) spatial dataset consisting of 4,620 polygon areas or records representing the boundary and overburden (feet) of the Pond Creek Coal Zone in the Appalachian Basin.
- **AppBasin_Pond_Creek_Coal_Zone_Thickness_USGS:** A (vector) spatial dataset consisting of 2,901 polygon areas or records representing the boundary and thickness (feet) of the Pond Creek Coal Zone in the Appalachian Basin.
- **AppBasin_Upper_Freeport_Coal_Bed_Elevation_USGS:** A (vector) spatial dataset consisting of 6,436 polygon areas or records representing the boundary and elevation above sea level (feet) of the top of the Upper Freeport Coal Bed in the Appalachian Basin.
- **AppBasin_Upper_Freeport_Coal_Bed_Overburden_USGS:** A (vector) spatial dataset consisting of 7,032 polygon areas or records representing the boundary and overburden (feet) of the Upper Freeport Coal Bed in the Appalachian Basin.
- **AppBasin_Upper_Freeport_Coal_Bed_Thickness_USGS:** A (vector) spatial dataset consisting of 9,334 polygon areas or records representing the boundary and thickness (feet) of the Upper Freeport Coal Bed in the Appalachian Basin.
- **Central_TX_Central_Coal_Zone_4_Overburden_USGS:** A (vector) spatial dataset consisting of 29 polygon areas or records representing the boundary and overburden (feet) of the Central Texas Central Coal Zone 4 in the Gulf Coast Region.
- **Central_TX_Central_Coal_Zone_4_Thickness_USGS:** A (vector) spatial dataset consisting of 31 polygon areas or records representing the boundary and thickness (feet) of the Central Texas Central Coal Zone 4 in the Gulf Coast Region.
- **Central_TX_Central_Coal_Zone_5_Overburden_USGS:** A (vector) spatial dataset consisting of 41 polygon areas or records representing the boundary and overburden (feet) of the Central Texas Central Coal Zone 5 in the Gulf Coast Region.
- **Central_TX_Central_Coal_Zone_5_Thickness_USGS:** A (vector) spatial dataset consisting of 24 polygon areas or records representing the boundary and thickness (feet) of the Central Texas Central Coal Zone 5 in the Gulf Coast Region.
- **Central_TX_North_Coal_Zone_5_Overburden_USGS:** A (vector) spatial dataset consisting of 257 polygon areas or records representing the boundary and overburden (feet) of the Central Texas North Coal Zone 5 in the Gulf Coast Region.
- **Central_TX_North_Coal_Zone_5_Thickness_USGS:** A (vector) spatial dataset consisting of 81 polygon areas or records representing the boundary and thickness (feet) of the Central Texas North Coal Zone 5 in the Gulf Coast Region.
- **Central_TX_North_Coal_Zone_6_Overburden_USGS:** A (vector) spatial dataset consisting of 118 polygon areas or records representing the boundary and overburden (feet) of the Central Texas North Coal Zone 6 in the Gulf Coast Region.
- **Central_TX_North_Coal_Zone_6_Thickness_USGS:** A (vector) spatial dataset consisting of 317 polygon areas or records representing the boundary and thickness (feet) of the Central Texas North Coal Zone 6 in the Gulf Coast Region.
- **Central_TX_North_Coal_Zone_8_Overburden_USGS:** A (vector) spatial dataset consisting of 265 polygon areas or records representing the boundary and overburden (feet) of the Central Texas North Coal Zone 8 in the Gulf Coast Region.
- **Central_TX_North_Coal_Zone_8_Thickness_USGS:** A (vector) spatial dataset consisting of 67 polygon areas or records representing the boundary and thickness (feet) of the Central Texas North Coal Zone 8 in the Gulf Coast Region.
- **Central_TX_North_Coal_Zone_9_Overburden_USGS:** A (vector) spatial dataset consisting of 160 polygon areas or records representing the boundary and overburden (feet) of the Central Texas North Coal Zone 9 in the Gulf Coast Region.
- **Central_TX_North_Coal_Zone_9_Thickness_USGS:** A (vector) spatial dataset consisting of 41 polygon areas or records representing the boundary and thickness (feet) of the Central Texas North Coal Zone 9 in the Gulf Coast Region.
- **Central_TX_South_Coal_Zone_4_Overburden_USGS:** A (vector) spatial dataset consisting of 74 polygon areas or records representing the boundary and overburden (feet) of the Central Texas South Coal Zone 4 in the Gulf Coast Region.
- **Central_TX_South_Coal_Zone_4_Thickness_USGS:** A (vector) spatial dataset consisting of 73 polygon areas or records representing the boundary and thickness (feet) of the Central Texas South Coal Zone 4 in the Gulf Coast Region.
- **Central_TX_South_Coal_Zone_6_Overburden_USGS:** A (vector) spatial dataset consisting of 139 polygon areas or records representing the boundary and overburden (feet) of the Central Texas South Coal Zone 6 in the Gulf Coast Region.
- **Central_TX_South_Coal_Zone_6_Thickness_USGS:** A (vector) spatial dataset consisting of 101 polygon areas or records representing the boundary and thickness (feet) of the Central Texas South Coal Zone 6 in the Gulf Coast Region.
- **Central_TX_South_Coal_Zone_8_Overburden_USGS:** A (vector) spatial dataset consisting of 77 polygon areas or records representing the boundary and overburden (feet) of the Central Texas South Coal Zone 8 in the Gulf Coast Region.
- **Central_TX_South_Coal_Zone_8_Thickness_USGS:** A (vector) spatial dataset consisting of 59 polygon areas or records representing the boundary and thickness (feet) of the Central Texas South Coal Zone 8 in the Gulf Coast Region.
- **CO_Plateau_Cameo_Wheeler_Coal_Field_Overburden_USGS:** A (vector) spatial dataset consisting of 5,305 polygon areas or records representing the boundary and overburden (feet) of the Cameo/Wheeler Coal Field in the Colorado Plateau Region.
- **CO_Plateau_Cameo_Wheeler_Coal_Field_Thickness_USGS:** A (vector) spatial dataset consisting of 96 polygon areas or records representing the boundary and thickness (feet) of the Cameo/Wheeler Coal Field in the Colorado Plateau Region.
- **CO_Plateau_Coal_Ridge_Coal_Field_Overburden_USGS:** A (vector) spatial dataset consisting of 2,418 polygon areas or records representing the boundary and overburden (feet) of the Coal Ridge Coal Field in the Colorado Plateau Region.
- **CO_Plateau_Coal_Ridge_Coal_Field_Thickness_USGS:** A (vector) spatial dataset consisting of 37 polygon areas or records representing the boundary and thickness (feet) of the Coal Ridge Coal Field in the Colorado Plateau Region.
- **CO_Plateau_Crested_Butte_Coal_Field_Overburden_USGS:** A (vector) spatial dataset consisting of 693 polygon areas or records representing the boundary and overburden (feet) of the Crested Butte Coal Field in the Colorado Plateau Region.
- **CO_Plateau_Crested_Butte_Coal_Field_Thickness_USGS:** A (vector) spatial dataset consisting of 22 polygon areas or records representing the boundary and thickness (feet) of the Crested Butte Coal Field in the Colorado Plateau Region.
- **CO_Plateau_Danforth_Hills_Coal_Field_Zone_A_Overburden_USGS:** A (vector) spatial dataset consisting of 1,524 polygon areas or records representing the boundary and overburden (feet) of Zone A of the Danforth Hills Coal Field in the Colorado Plateau Region.
- **CO_Plateau_Danforth_Hills_Coal_Field_Zone_A_Thickness_USGS:** A (vector) spatial dataset consisting of 36 polygon areas or records representing the boundary and thickness (feet) of Zone A of the Danforth Hills Coal Field in the Colorado Plateau Region.
- **CO_Plateau_Danforth_Hills_Coal_Field_Zone_B_Overburden_USGS:** A (vector) spatial dataset consisting of 1,339 polygon areas or records representing the boundary and overburden (feet) of Zone B of the Danforth Hills Coal Field in the Colorado Plateau Region.
- **CO_Plateau_Danforth_Hills_Coal_Field_Zone_B_Thickness_USGS:** A (vector) spatial dataset consisting of 72 polygon areas or records representing the boundary and thickness (feet) of Zone B of the Danforth Hills Coal Field in the Colorado Plateau Region.
- **CO_Plateau_Danforth_Hills_Coal_Field_Zone_C_Overburden_USGS:** A (vector) spatial dataset consisting of 1,364 polygon areas or records representing the boundary and overburden (feet) of Zone C of the Danforth Hills Coal Field in the Colorado Plateau Region.
- **CO_Plateau_Danforth_Hills_Coal_Field_Zone_C_Thickness_USGS:** A (vector) spatial dataset consisting of 63 polygon areas or records representing the boundary and thickness (feet) of Zone C of the Danforth Hills Coal Field in the Colorado Plateau Region.
- **CO_Plateau_Danforth_Hills_Coal_Field_Zone_D_Overburden_USGS:** A (vector) spatial dataset consisting of 1,373 polygon areas or records representing the boundary and overburden (feet) of Zone D of the Danforth Hills Coal Field in the Colorado Plateau Region.
- **CO_Plateau_Danforth_Hills_Coal_Field_Zone_D_Thickness_USGS:** A (vector) spatial dataset consisting of 58 polygon areas or records representing the boundary and thickness (feet) of Zone D of the Danforth Hills Coal Field in the Colorado Plateau Region.
- **CO_Plateau_Danforth_Hills_Coal_Field_Zone_E_Overburden_USGS:** A (vector) spatial dataset consisting of 1,094 polygon areas or records representing the boundary and overburden (feet) of Zone E of the Danforth Hills Coal Field in the Colorado Plateau Region.
- **CO_Plateau_Danforth_Hills_Coal_Field_Zone_E_Thickness_USGS:** A (vector) spatial dataset consisting of 189 polygon areas or records representing the boundary and thickness (feet) of Zone E of the Danforth Hills Coal Field in the Colorado Plateau Region.
- **CO_Plateau_Danforth_Hills_Coal_Field_Zone_F_Overburden_USGS:** A (vector) spatial dataset consisting of 1,037 polygon areas or records representing the boundary and overburden (feet) of Zone F of the Danforth Hills Coal Field in the Colorado Plateau Region.
- **CO_Plateau_Danforth_Hills_Coal_Field_Zone_F_Thickness_USGS:** A (vector) spatial dataset consisting of 105 polygon areas or records representing the boundary and thickness (feet) of Zone F of the Danforth Hills Coal Field in the Colorado Plateau Region.
- **CO_Plateau_Danforth_Hills_Coal_Field_Zone_G_Overburden_USGS:** A (vector) spatial dataset consisting of 812 polygon areas or records representing the boundary and overburden (feet) of Zone G of the Danforth Hills Coal Field in the Colorado Plateau Region.
- **CO_Plateau_Danforth_Hills_Coal_Field_Zone_G_Thickness_USGS:** A (vector) spatial dataset consisting of 45 polygon areas or records representing the boundary and thickness (feet) of Zone G of the Danforth Hills Coal Field in the Colorado Plateau Region.
- **CO_Plateau_Kaiparowits_Coal_Field_Overburden_USGS:** A (vector) spatial dataset consisting of 5,222 polygon areas or records representing the boundary and overburden (feet) of the Kaiparowitz Coal Field in the Colorado Plateau Region.
- **CO_Plateau_Kaiparowits_Coal_Field_Thickness_USGS:** A (vector) spatial dataset consisting of 4,024 polygon areas or records representing the boundary and thickness (feet) of the Kaiparowitz Coal Field in the Colorado Plateau Region.
- **CO_Plateau_Lower_White_River_Coal_Field_Zone_B_Overburden_USGS:** A (vector) spatial dataset consisting of 99 polygon areas or records representing the boundary and overburden (feet) of Zone B of the Lower White River Coal Field in the Colorado Plateau Region.
- **CO_Plateau_Lower_White_River_Coal_Field_Zone_B_Thickness_USGS:** A (vector) spatial dataset consisting of 37 polygon areas or records representing the boundary and thickness (feet) of Zone B of the Lower White River Coal Field in the Colorado Plateau Region.
- **CO_Plateau_Lower_White_River_Coal_Field_Zone_D_Overburden_USGS:** A (vector) spatial dataset consisting of 117 polygon areas or records representing the boundary and overburden (feet) of Zone D of the Lower White River Coal Field in the Colorado Plateau Region.
- **CO_Plateau_Lower_White_River_Coal_Field_Zone_D_Thickness_USGS:** A (vector) spatial dataset consisting of 25 polygon areas or records representing the boundary and thickness (feet) of Zone D of the Lower White River Coal Field in the Colorado Plateau Region.
- **CO_Plateau_San_Juan_Basin_Overburden_USGS:** A (vector) spatial dataset consisting of 7,284 polygon areas or records representing the boundary and overburden (feet) of the San Juan Basin in the Colorado Plateau Region.
- **CO_Plateau_San_Juan_Basin_Thickness_USGS:** A (vector) spatial dataset consisting of 90 polygon areas or records representing the boundary and thickness (feet) of the San Juan Basin in the Colorado Plateau Region.
- **CO_Plateau_South_Canyon_Coal_Field_Overburden_USGS:** A (vector) spatial dataset consisting of 2,340 polygon areas or records representing the boundary and overburden (feet) of the South Canyon Coal Field in the Colorado Plateau Region.
- **CO_Plateau_South_Canyon_Coal_Field_Thickness_USGS:** A (vector) spatial dataset consisting of 48 polygon areas or records representing the boundary and thickness (feet) of the South Canyon Coal Field in the Colorado Plateau Region.
- **CO_Plateau_South_Piceance_Coal_Field_Thickness_USGS:** A (vector) spatial dataset consisting of 68 polygon areas or records representing the boundary and thickness (feet) of the Piceance Coal Field in the Colorado Plateau Region.
- **CO_Plateau_South_Wasatch_Plateau_Coal_Field_Overburden_USGS:** A (vector) spatial dataset consisting of 865 polygon areas or records representing the boundary and overburden (feet) of the South Wasatch Plateau Coal Field in the Colorado Plateau Region.
- **CO_Plateau_South_Wasatch_Plateau_Coal_Field_Thickness_USGS:** A (vector) spatial dataset consisting of 81 polygon areas or records representing the boundary and thickness (feet) of the South Wasatch Plateau Coal Field in the Colorado Plateau Region.
- **CO_Plateau_Yampa_Coal_Field_Zone_A_Overburden_USGS:** A (vector) spatial dataset consisting of 1,421 polygon areas or records representing the boundary and overburden (feet) of Zone A of the Yampa Coal Field in the Colorado Plateau Region.
- **CO_Plateau_Yampa_Coal_Field_Zone_A_Thickness_USGS:** A (vector) spatial dataset consisting of 22 polygon areas or records representing the boundary and thickness (feet) of Zone A of the Yampa Coal Field in the Colorado Plateau Region.
- **CO_Plateau_Yampa_Coal_Field_Zone_B_Overburden_USGS:** A (vector) spatial dataset consisting of 900 polygon areas or records representing the boundary and overburden (feet) of Zone B of the Yampa Coal Field in the Colorado Plateau Region.
- **CO_Plateau_Yampa_Coal_Field_Zone_B_Thickness_USGS:** A (vector) spatial dataset consisting of 19 polygon areas or records representing the boundary and thickness (feet) of Zone B of the Yampa Coal Field in the Colorado Plateau Region.
- **CO_Plateau_Yampa_Coal_Field_Zone_C_Overburden_USGS:** A (vector) spatial dataset consisting of 956 polygon areas or records representing the boundary and overburden (feet) of Zone C of the Yampa Coal Field in the Colorado Plateau Region.
- **CO_Plateau_Yampa_Coal_Field_Zone_C_Thickness_USGS:** A (vector) spatial dataset consisting of 31 polygon areas or records representing the boundary and thickness (feet) of Zone C of the Yampa Coal Field in the Colorado Plateau Region.
- **CO_Plateau_Yampa_Coal_Field_Zone_D_Overburden_USGS:** A (vector) spatial dataset consisting of 848 polygon areas or records representing the boundary and overburden (feet) of Zone D of the Yampa Coal Field in the Colorado Plateau Region.
- **CO_Plateau_Yampa_Coal_Field_Zone_D_Thickness_USGS:** A (vector) spatial dataset consisting of 20 polygon areas or records representing the boundary and thickness (feet) of Zone D of the Yampa Coal Field in the Colorado Plateau Region.
- **Coal_fields_USGS:** A (vector) spatial dataset consisting of 602 polygon areas or records representing coal fields and regions within the United States.
- **Coal_stratigraphy_USTRAT_USGS:** A table consisting of 517,104 records representing coal stratigraphic attributes and measurements in the United States.
- **Coal_stratigraphy_metadata_USTRAT_USGS:** A (vector) spatial dataset consisting of 253,984 point locations and metadata records related to the “Coal_stratigraphy_USTRAT_USGS” table dataset.
- **Green_River_Basin_Black_Butte_Deadman_Coal_Zone_Overburden_USGS:** A (vector) spatial dataset consisting of 51 polygon areas or records representing the boundary and overburden (feet) of the Black Butte/Deadman Coal Zone in the Green River Basin.
- **Green_River_Basin_Black_Butte_Deadman_Coal_Zone_Thickness_USGS:** A (vector) spatial dataset consisting of 104 polygon areas or records representing the boundary and thickness (feet) of the Black Butte/Deadman Coal Zone in the Green River Basin.
- **Green_River_Basin_Jim_Bridger_Deadman_Coal_Zone_Overburden_USGS:** A (vector) spatial dataset consisting of 43 polygon areas or records representing the boundary and overburden (feet) of the Jim Bridger/Deadman Coal Zone in the Green River Basin.
- **Green_River_Basin_Jim_Bridger_Deadman_Coal_Zone_Thickness_USGS:** A (vector) spatial dataset consisting of 86 polygon areas or records representing the boundary and thickness (feet) of the Jim Bridger/Deadman Coal Zone in the Green River Basin.
- **Hannah_Basin_Ferris_23_Coal_Zone_Overburden_USGS:** A (vector) spatial dataset consisting of 413 polygon areas or records representing the boundary and overburden (feet) of the Ferris 23 Coal Zone in the Hannah Basin.
- **Hannah_Basin_Ferris_23_Coal_Zone_Thickness_USGS:** A (vector) spatial dataset consisting of 16 polygon areas or records representing the boundary and thickness (feet) of the Ferris 23 Coal Zone in the Hannah Basin.
- **Hannah_Basin_Ferris_25_Coal_Zone_Overburden_USGS:** A (vector) spatial dataset consisting of 462 polygon areas or records representing the boundary and overburden (feet) of the Ferris 25 Coal Zone in the Hannah Basin.
- **Hannah_Basin_Ferris_25_Coal_Zone_Thickness_USGS:** A (vector) spatial dataset consisting of 22 polygon areas or records representing the boundary and thickness (feet) of the Ferris 25 Coal Zone in the Hannah Basin.
- **Hannah_Basin_Ferris_31_Coal_Zone_Overburden_USGS:** A (vector) spatial dataset consisting of 298 polygon areas or records representing the boundary and overburden (feet) of the Ferris 31 Coal Zone in the Hannah Basin.
- **Hannah_Basin_Ferris_31_Coal_Zone_Thickness_USGS:** A (vector) spatial dataset consisting of 17 polygon areas or records representing the boundary and thickness (feet) of the Ferris 31 Coal Zone in the Hannah Basin.
- **Hannah_Basin_Ferris_50_Coal_Zone_Overburden_USGS:** A (vector) spatial dataset consisting of 213 polygon areas or records representing the boundary and overburden (feet) of the Ferris 50 Coal Zone in the Hannah Basin.
- **Hannah_Basin_Ferris_50_Coal_Zone_Thickness_USGS:** A (vector) spatial dataset consisting of 13 polygon areas or records representing the boundary and thickness (feet) of the Ferris 50 Coal Zone in the Hannah Basin.
- **Hannah_Basin_Ferris_65_Coal_Zone_Overburden_USGS:** A (vector) spatial dataset consisting of 54 polygon areas or records representing the boundary and overburden (feet) of the Ferris 65 Coal Zone in the Hannah Basin.
- **Hannah_Basin_Ferris_65_Coal_Zone_Thickness_USGS:** A (vector) spatial dataset consisting of 26 polygon areas or records representing the boundary and thickness (feet) of the Ferris 65 Coal Zone in the Hannah Basin.
- **Hannah_Basin_Hannah_77_Coal_Zone_Overburden_USGS:** A (vector) spatial dataset consisting of 42 polygon areas or records representing the boundary and overburden (feet) of the Hannah 77 Coal Zone in the Hannah Basin.
- **Hannah_Basin_Hannah_77_Coal_Zone_Thickness_USGS:** A (vector) spatial dataset consisting of 25 polygon areas or records representing the boundary and thickness (feet) of the Hannah 77 Coal Zone in the Hannah Basin.
- **Hannah_Basin_Hannah_78_Coal_Zone_Overburden_USGS:** A (vector) spatial dataset consisting of 35 polygon areas or records representing the boundary and overburden (feet) of the Hannah 78 Coal Zone in the Hannah Basin.
- **Hannah_Basin_Hannah_78_Coal_Zone_Thickness_USGS:** A (vector) spatial dataset consisting of 23 polygon areas or records representing the boundary and thickness (feet) of the Hannah 78 Coal Zone in the Hannah Basin.
- **Hannah_Basin_Hannah_79_Coal_Zone_Overburden_USGS:** A (vector) spatial dataset consisting of 20 polygon areas or records representing the boundary and overburden (feet) of the Hannah 79 Coal Zone in the Hannah Basin.
- **Hannah_Basin_Hannah_79_Coal_Zone_Thickness_USGS:** A (vector) spatial dataset consisting of 25 polygon areas or records representing the boundary and thickness (feet) of the Hannah 79 Coal Zone in the Hannah Basin.
- **Hannah_Basin_Hannah_81_Coal_Zone_Overburden_USGS:** A (vector) spatial dataset consisting of 26 polygon areas or records representing the boundary and overburden (feet) of the Hannah 81 Coal Zone in the Hannah Basin.
- **Hannah_Basin_Hannah_81_Coal_Zone_Thickness_USGS:** A (vector) spatial dataset consisting of 19 polygon areas or records representing the boundary and thickness (feet) of the Hannah 81 Coal Zone in the Hannah Basin.
- **Hannah_Basin_Johnson_107_Coal_Zone_Overburden_USGS:** A (vector) spatial dataset consisting of 13 polygon areas or records representing the boundary and overburden (feet) of the Johnson 107 Coal Zone in the Hannah Basin.
- **Hannah_Basin_Johnson_107_Coal_Zone_Thickness_USGS:** A (vector) spatial dataset consisting of 28 polygon areas or records representing the boundary and thickness (feet) of the Johnson 107 Coal Zone in the Hannah Basin.
- **IL_Basin_Baker_Danville_Coal_Overburden_USGS:** A (vector) spatial dataset consisting of 352 polygon areas or records representing the boundary and overburden (feet) of the Baker/Danville Coal Bed in the Illinois Basin.
- **IL_Basin_Baker_Danville_Coal_Thickness_USGS:** A (vector) spatial dataset consisting of 1,030 polygon areas or records representing the boundary and thickness (feet) of the Baker/Danville Coal Bed in the Illinois Basin.
- **IL_Basin_Herrin_Coal_Overburden_USGS:** A (vector) spatial dataset consisting of 305 polygon areas or records representing the boundary and overburden (feet) of the Herrin Coal Bed in the Illinois Basin.
- **IL_Basin_Herrin_Coal_Thickness_USGS:** A (vector) spatial dataset consisting of 1,359 polygon areas or records representing the boundary and thickness (feet) of the Herrin Coal Bed in the Illinois Basin.
- **IL_Basin_Springfield_Coal_Overburden_USGS:** A (vector) spatial dataset consisting of 4 polygon areas or records representing the boundary and overburden (feet) of the Springfield Coal Bed in the Illinois Basin.
- **IL_Basin_Springfield_Coal_Thickness_USGS:** A (vector) spatial dataset consisting of 1,136 polygon areas or records representing the boundary and thickness (feet) of the Springfield Coal Bed in the Illinois Basin.
- **IL_Colchester_Avail_SF_Py_ISGS:** A (vector) spatial dataset consisting of 8,726 polygon areas or records representing restricted or available surface coal mining areas for the Colchester Coal Bed in Illinois.
- **IL_Colchester_Avail_UG_Py_ISGS:** A (vector) spatial dataset consisting of 2,338 polygon areas or records representing restricted or available underground coal mining areas for the Colchester Coal Bed in Illinois.
- **IL_Colchester_Crop_Py_ISGS:** A (vector) spatial dataset consisting of 76 polygon areas or records representing subcrop of the Colchester Coal Bed in Illinois.
- **IL_Colchester_Depth_Ln_ISGS:** A (vector) spatial dataset consisting of 216 polyline contours or records representing depth to the top (feet) of the Colchester Coal Bed in Illinois.
- **IL_Colchester_Depth_Py_ISGS:** A (vector) spatial dataset consisting of 261 polygon areas or records representing depth to the top (feet) of the Colchester Coal Bed in Illinois.
- **IL_Colchester_Elevation_Ln_ISGS:** A (vector) spatial dataset consisting of 8,192 polyline contours or records representing the boundary and elevation above sea level (feet) of the top of the Colchester Coal Bed in Illinois.
- **IL_Colchester_Elevation_Py_ISGS:** A (vector) spatial dataset consisting of 2,642 polygon areas or records representing the boundary and elevation above sea level (feet) of the top of the Colchester Coal Bed in Illinois.
- **IL_Colchester_Thickness_Py_ISGS:** A (vector) spatial dataset consisting of 302 polygon areas or records representing the boundary and thickness (inches) of the Colchester Coal Bed in Illinois.
- **IL_Danville_Avail_SF_Py_ISGS:** A (vector) spatial dataset consisting of 2,699 polygon areas or records representing restricted or available surface coal mining areas for the Danville Coal Bed in Illinois.
- **IL_Danville_Avail_UG_Py_ISGS:** A (vector) spatial dataset consisting of 2,426 polygon areas or records representing restricted or available underground coal mining areas for the Danville Coal Bed in Illinois.
- **IL_Danville_Crop_Py_ISGS:** A (vector) spatial dataset consisting of 37 polygon areas or records representing subcrop of the Danville Coal Bed in Illinois.
- **IL_Danville_Depth_Ln_ISGS:** A (vector) spatial dataset consisting of 280 polyline contours or records representing depth to the top (feet) of the Danville Coal Bed in Illinois.
- **IL_Danville_Depth_Py_ISGS:** A (vector) spatial dataset consisting of 112 polygon areas or records representing depth to the top (feet) of the Danville Coal Bed in Illinois.
- **IL_Danville_Elevation_Ln_ISGS:** A (vector) spatial dataset consisting of 282 polyline contours or records representing the boundary and elevation above sea level (feet) of the top of the Danville Coal Bed in Illinois.
- **IL_Danville_Elevation_Py_ISGS:** A (vector) spatial dataset consisting of 116 polygon areas or records representing the boundary and elevation above sea level (feet) of the top of the Danville Coal Bed in Illinois.
- **IL_Danville_Thickness_Py_ISGS:** A (vector) spatial dataset consisting of 416 polygon areas or records representing the boundary and thickness (inches) of the Danville Coal Bed in Illinois.
- **IL_Davis_Avail_UG_Py_ISGS:** A (vector) spatial dataset consisting of 1,477 polygon areas or records representing restricted or available underground coal mining areas for the Davis Coal Bed in Illinois.
- **IL_Davis_Depth_Ln_ISGS:** A (vector) spatial dataset consisting of 163 polyline contours or records representing depth to the top (feet) of the Davis Coal Bed in Illinois.
- **IL_Davis_Depth_Py_ISGS:** A (vector) spatial dataset consisting of 85 polygon areas or records representing depth to the top (feet) of the Davis Coal Bed in Illinois.
- **IL_Davis_Thickness_Py_ISGS:** A (vector) spatial dataset consisting of 257 polygon areas or records representing the boundary and thickness (inches) of the Davis Coal Bed in Illinois.
- **IL_DekovDavis_Avail_SF_Py_ISGS:** A (vector) spatial dataset consisting of 189 polygon areas or records representing restricted or available surface coal mining areas for the Dekoven and Davis Coal Beds in Illinois.
- **IL_Dekoven_Avail_UG_Py_ISGS:** A (vector) spatial dataset consisting of 1,233 polygon areas or records representing restricted or available underground coal mining areas for the Dekoven Coal Bed in Illinois.
- **IL_Dekoven_Thickness_Py_ISGS:** A (vector) spatial dataset consisting of 277 polygon areas or records representing the boundary and thickness (inches) of the Dekoven Coal Bed in Illinois.
- **IL_Herrin_Avail_SF_Py_ISGS:** A (vector) spatial dataset consisting of 9,220 polygon areas or records representing restricted or available surface coal mining areas for the Herrin Coal Bed in Illinois.
- **IL_Herrin_Avail_UG_Py_ISGS:** A (vector) spatial dataset consisting of 10,451 polygon areas or records representing restricted or available underground coal mining areas for the Herrin Coal Bed in Illinois.
- **IL_Herrin_Crop_Py_ISGS:** A (vector) spatial dataset consisting of 87 polygon areas or records representing subcrop of the Herrin Coal Bed in Illinois.
- **IL_Herrin_Depth_Ln_ISGS:** A (vector) spatial dataset consisting of 7,962 polyline contours or records representing depth to the top (feet) of the Herrin Coal Bed in Illinois.
- **IL_Herrin_Depth_Py_ISGS:** A (vector) spatial dataset consisting of 3,904 polygon areas or records representing depth to the top (feet) of the Herrin Coal Bed in Illinois.
- **IL_Herrin_Elevation_Ln_ISGS:** A (vector) spatial dataset consisting of 7,374 polyline contours or records representing the boundary and elevation above sea level (feet) of the top of the Herrin Coal Bed in Illinois.
- **IL_Herrin_Elevation_Py_ISGS:** A (vector) spatial dataset consisting of 3,130 polygon areas or records representing the boundary and elevation above sea level (feet) of the top of the Herrin Coal Bed in Illinois.
- **IL_Herrin_Thickness_Py_ISGS:** A (vector) spatial dataset consisting of 853 polygon areas or records representing the boundary and thickness (inches) of the Herrin Coal Bed in Illinois.
- **IL_Jamestown_Avail_SF_Py_ISGS:** A (vector) spatial dataset consisting of 27 polygon areas or records representing restricted or available surface coal mining areas for the Jamestown Coal Bed in Illinois.
- **IL_Jamestown_Avail_UG_Py_ISGS:** A (vector) spatial dataset consisting of 373 polygon areas or records representing restricted or available underground coal mining areas for the Jamestown Coal Bed in Illinois.
- **IL_Jamestown_Depth_Ln_ISGS:** A (vector) spatial dataset consisting of 115 polyline contours or records representing depth to the top (feet) of the Jamestown Coal Bed in Illinois.
- **IL_Jamestown_Depth_Py_ISGS:** A (vector) spatial dataset consisting of 88 polygon areas or records representing depth to the top (feet) of the Jamestown Coal Bed in Illinois.
- **IL_Jamestown_Elevation_Ln_ISGS:** A (vector) spatial dataset consisting of 47 polyline contours or records representing the boundary and elevation above sea level (feet) of the top of the Jamestown Coal Bed in Illinois.
- **IL_Jamestown_Elevation_Py_ISGS:** A (vector) spatial dataset consisting of 49 polygon areas or records representing the boundary and elevation above sea level (feet) of the top of the Jamestown Coal Bed in Illinois.
- **IL_Jamestown_Thickness_Py_ISGS:** A (vector) spatial dataset consisting of 45 polygon areas or records representing the boundary and thickness (inches) of the Jamestown Coal Bed in Illinois.
- **IL_Net_Coal_Thickness_Py_ISGS:** A (vector) spatial dataset consisting of 1,235 polygon areas or records representing the boundary and net cumulative thickness (feet) of Coal in Illinois.
- **IL_Seelyville_Avail_SF_Py_ISGS:** A (vector) spatial dataset consisting of 14 polygon areas or records representing restricted or available surface coal mining areas for the Seelyville Coal Bed in Illinois.
- **IL_Seelyville_Avail_UG_Py_ISGS:** A (vector) spatial dataset consisting of 596 polygon areas or records representing restricted or available underground coal mining areas for the Seelyville Coal Bed in Illinois.
- **IL_Seelyville_Depth_Ln_ISGS:** A (vector) spatial dataset consisting of 139 polyline contours or records representing depth to the top (feet) of the Seelyville Coal Bed in Illinois.
- **IL_Seelyville_Depth_Py_ISGS:** A (vector) spatial dataset consisting of 50 polygon areas or records representing depth to the top (feet) of the Seelyville Coal Bed in Illinois.
- **IL_Seelyville_Elevation_Ln_ISGS:** A (vector) spatial dataset consisting of 69 polyline contours or records representing the boundary and elevation above sea level (feet) of the top of the Seelyville Coal Bed in Illinois.
- **IL_Seelyville_Elevation_Py_ISGS:** A (vector) spatial dataset consisting of 73 polygon areas or records representing the boundary and elevation above sea level (feet) of the top of the Seelyville Coal Bed in Illinois.
- **IL_Seelyville_Thickness_Py_ISGS:** A (vector) spatial dataset consisting of 69 polygon areas or records representing the boundary and thickness (inches) of the Seelyville Coal Bed in Illinois.
- **IL_Springfield_Avail_SF_Py_ISGS:** A (vector) spatial dataset consisting of 5,475 polygon areas or records representing restricted or available surface coal mining areas for the Springfield Coal Bed in Illinois.
- **IL_Springfield_Avail_UG_Py_ISGS:** A (vector) spatial dataset consisting of 6,745 polygon areas or records representing restricted or available underground coal mining areas for the Springfield Coal Bed in Illinois.
- **IL_Springfield_Crop_Py_ISGS:** A (vector) spatial dataset consisting of 42 polygon areas or records representing subcrop of the Springfield Coal Bed in Illinois.
- **IL_Springfield_Depth_Ln_ISGS:** A (vector) spatial dataset consisting of 6,846 polyline contours or records representing depth to the top (feet) of the Springfield Coal Bed in Illinois.
- **IL_Springfield_Depth_Py_ISGS:** A (vector) spatial dataset consisting of 3,089 polygon areas or records representing depth to the top (feet) of the Springfield Coal Bed in Illinois.
- **IL_Springfield_Elevation_Ln_ISGS:** A (vector) spatial dataset consisting of 5,678 polyline contours or records representing the boundary and elevation above sea level (feet) of the top of the Springfield Coal Bed in Illinois.
- **IL_Springfield_Elevation_Py_ISGS:** A (vector) spatial dataset consisting of 2,397 polygon areas or records representing the boundary and elevation above sea level (feet) of the top of the Springfield Coal Bed in Illinois.
- **IL_Springfield_Thickness_Py_ISGS:** A (vector) spatial dataset consisting of 702 polygon areas or records representing the boundary and thickness (inches) of the Springfield Coal Bed in Illinois.
- **LA_Sabine_Overburden_USGS:** A (vector) spatial dataset consisting of 293 polygon areas or records representing the boundary and overburden (feet) of the Louisiana Sabine Coal Bed in the Gulf Coast Region.
- **LA_Sabine_Thickness_USGS:** A (vector) spatial dataset consisting of 154 polygon areas or records representing the boundary and thickness (feet) of the Louisiana Sabine Coal Bed in the Gulf Coast Region.
- **Northeast_TX_Coal_Zone_1_Overburden_USGS:** A (vector) spatial dataset consisting of 208 polygon areas or records representing the boundary and overburden (feet) of Zone 1 in Northeast Texas in the Gulf Coast Region.
- **Northeast_TX_Coal_Zone_1_Thickness_USGS:** A (vector) spatial dataset consisting of 817 polygon areas or records representing the boundary and thickness (feet) of Zone 1 in Northeast Texas in the Gulf Coast Region.
- **Northeast_TX_Coal_Zone_2_Overburden_USGS:** A (vector) spatial dataset consisting of 274 polygon areas or records representing the boundary and overburden (feet) of Zone 2 in Northeast Texas in the Gulf Coast Region.
- **Northeast_TX_Coal_Zone_2_Thickness_USGS:** A (vector) spatial dataset consisting of 1,276 polygon areas or records representing the boundary and thickness (feet) of Zone 2 in Northeast Texas in the Gulf Coast Region.
- **Northeast_TX_Coal_Zone_3_Overburden_USGS:** A (vector) spatial dataset consisting of 225 polygon areas or records representing the boundary and overburden (feet) of Zone 3 in Northeast Texas in the Gulf Coast Region.
- **Northeast_TX_Coal_Zone_3_Thickness_USGS:** A (vector) spatial dataset consisting of 1,401 polygon areas or records representing the boundary and thickness (feet) of Zone 3 in Northeast Texas in the Gulf Coast Region.
- **Northeast_TX_Coal_Zone_4_Overburden_USGS:** A (vector) spatial dataset consisting of 206 polygon areas or records representing the boundary and overburden (feet) of Zone 4 in Northeast Texas in the Gulf Coast Region.
- **Northeast_TX_Coal_Zone_4_Thickness_USGS:** A (vector) spatial dataset consisting of 1,780 polygon areas or records representing the boundary and thickness (feet) of Zone 4 in Northeast Texas in the Gulf Coast Region.
- **Northeast_TX_Coal_Zone_5_Overburden_USGS:** A (vector) spatial dataset consisting of 143 polygon areas or records representing the boundary and overburden (feet) of Zone 5 in Northeast Texas in the Gulf Coast Region.
- **Northeast_TX_Coal_Zone_5_Thickness_USGS:** A (vector) spatial dataset consisting of 1,453 polygon areas or records representing the boundary and thickness (feet) of Zone 5 in Northeast Texas in the Gulf Coast Region.
- **Northeast_TX_Coal_Zone_6_Overburden_USGS:** A (vector) spatial dataset consisting of 1,133 polygon areas or records representing the boundary and overburden (feet) of Zone 6 in Northeast Texas in the Gulf Coast Region.
- **Northeast_TX_Coal_Zone_6_Thickness_USGS:** A (vector) spatial dataset consisting of 59 polygon areas or records representing the boundary and thickness (feet) of Zone 6 in Northeast Texas in the Gulf Coast Region.
- **OK_Coal_stratigraphic_data_OGS:** A table consisting of 25,518 records representing coal stratigraphic attributes and measurements in Oklahoma.
- **OK_Coal_stratigraphic_metadata_OGS:** A table consisting of 4,496 point locations and metadata records related to the “OK_COAL_stratigraphic_data_OGS” table dataset.
- **Powder_River_Basin_A_Zone_Coal_Bed_Overburden_USGS:** A (raster) spatial dataset representing the boundary and overburden (feet) of the A Zone Coal Bed in the Powder River Basin.
- **Powder_River_Basin_A_Zone_Coal_Bed_Reliability_USGS:** A (raster) spatial dataset representing the reliability of the A Zone Coal Bed measurements, categorized by *measured*, *indicated*, *inferred*, or *hypothetical*.
- **Powder_River_Basin_A_Zone_Coal_Bed_Thickness_USGS:** A (raster) spatial dataset representing the boundary and thickness (feet) of the A Zone Coal Bed in the Powder River Basin.
- **Powder_River_Basin_Anderson_Coal_Bed_Overburden_USGS:** A (raster) spatial dataset representing the boundary and overburden (feet) of the Anderson Coal Bed in the Powder River Basin.
- **Powder_River_Basin_Anderson_Coal_Bed_Reliability_USGS:** A (raster) spatial dataset representing the reliability of the Anderson Coal Bed measurements, categorized by *measured*, *indicated*, *inferred*, or *hypothetical*.
- **Powder_River_Basin_Anderson_Coal_Bed_Thickness_USGS:** A (raster) spatial dataset representing the boundary and thickness (feet) of the Anderson Coal Bed in the Powder River Basin.
- **Powder_River_Basin_Anderson_Lower_Rider_Coal_Bed_Overburden_USGS:** A (raster) spatial dataset representing the boundary and overburden (feet) of the Anderson Lower Rider Coal Bed in the Powder River Basin.
- **Powder_River_Basin_Anderson_Lower_Rider_Coal_Bed_Reliability_USGS:** A (raster) spatial dataset representing the reliability of the Anderson Lower Rider Coal Bed measurements, categorized by *measured*, *indicated*, *inferred*, or *hypothetical*.
- **Powder_River_Basin_Anderson_Lower_Rider_Coal_Bed_Thickness_USGS:** A (raster) spatial dataset representing the boundary and thickness (feet) of the Anderson Lower Rider Coal Bed in the Powder River Basin.
- **Powder_River_Basin_Anderson_Upper_Rider_Coal_Bed_Overburden_USGS:** A (raster) spatial dataset representing the boundary and overburden (feet) of the Anderson Upper Rider Coal Bed in the Powder River Basin.
- **Powder_River_Basin_Anderson_Upper_Rider_Coal_Bed_Reliability_USGS:** A (raster) spatial dataset representing the reliability of the Anderson Upper Rider Coal Bed measurements, categorized by *measured*, *indicated*, *inferred*, or *hypothetical*.
- **Powder_River_Basin_Anderson_Upper_Rider_Coal_Bed_Thickness_USGS:** A (raster) spatial dataset representing the boundary and thickness (feet) of the Anderson Upper Rider Coal Bed in the Powder River Basin.
- **Powder_River_Basin_Ashland_Coal_Field_Overburden_USGS:** A (vector) spatial dataset consisting of 344 polygon areas or records representing the boundary and overburden (feet) of the Ashland Coal Field in the Powder River Basin.
- **Powder_River_Basin_Ashland_Coal_Field_Thickness_USGS:** A (vector) spatial dataset consisting of 7 polygon areas or records representing the boundary and thickness (feet) of the Ashland Coal Field in the Powder River Basin.
- **Powder_River_Basin_Brewster_Arnold_Coal_Bed_Overburden_USGS:** A (raster) spatial dataset representing the boundary and overburden (feet) of the Brewster-Arnold Coal Bed in the Powder River Basin.
- **Powder_River_Basin_Brewster_Arnold_Coal_Bed_Reliability_USGS:** A (raster) spatial dataset representing the reliability of the Brewster-Arnold Coal Bed measurements, categorized by *measured*, *indicated*, *inferred*, or *hypothetical*.
- **Powder_River_Basin_Brewster_Arnold_Coal_Bed_Thickness_USGS:** A (raster) spatial dataset representing the boundary and thickness (feet) of the Brewster-Arnold Coal Bed in the Powder River Basin.
- **Powder_River_Basin_Burley_Coal_Bed_Overburden_USGS:** A (raster) spatial dataset representing the boundary and overburden (feet) of the Burley Coal Bed in the Powder River Basin.
- **Powder_River_Basin_Burley_Coal_Bed_Reliability_USGS:** A (raster) spatial dataset representing the reliability of the Burley Coal Bed measurements, categorized by *measured*, *indicated*, *inferred*, or *hypothetical*.
- **Powder_River_Basin_Burley_Coal_Bed_Thickness_USGS:** A (raster) spatial dataset representing the boundary and thickness (feet) of the Burley Coal Bed in the Powder River Basin.
- **Powder_River_Basin_Cache_Coal_Bed_Overburden_USGS:** A (raster) spatial dataset representing the boundary and overburden (feet) of the Cache Coal Bed in the Powder River Basin.
- **Powder_River_Basin_Cache_Coal_Bed_Reliability_USGS:** A (raster) spatial dataset representing the reliability of the Cache Coal Bed measurements, categorized by *measured*, *indicated*, *inferred*, or *hypothetical*.
- **Powder_River_Basin_Cache_Coal_Bed_Thickness_USGS:** A (raster) spatial dataset representing the boundary and thickness (feet) of the Cache Coal Bed in the Powder River Basin.
- **Powder_River_Basin_Calvert_Coal_Bed_Overburden_USGS:** A (raster) spatial dataset representing the boundary and overburden (feet) of the Calvert Coal Bed in the Powder River Basin.
- **Powder_River_Basin_Calvert_Coal_Bed_Reliability_USGS:** A (raster) spatial dataset representing the reliability of the Calvert Coal Bed measurements, categorized by *measured*, *indicated*, *inferred*, or *hypothetical*.
- **Powder_River_Basin_Calvert_Coal_Bed_Thickness_USGS:** A (raster) spatial dataset representing the boundary and thickness (feet) of the Calvert Coal Bed in the Powder River Basin.
- **Powder_River_Basin_Canyon_Coal_Bed_Overburden_USGS:** A (raster) spatial dataset representing the boundary and overburden (feet) of the Canyon Coal Bed in the Powder River Basin.
- **Powder_River_Basin_Canyon_Coal_Bed_Reliability_USGS:** A (raster) spatial dataset representing the reliability of the Canyon Coal Bed measurements, categorized by *measured*, *indicated*, *inferred*, or *hypothetical*.
- **Powder_River_Basin_Canyon_Coal_Bed_Thickness_USGS:** A (raster) spatial dataset representing the boundary and thickness (feet) of the Canyon Coal Bed in the Powder River Basin.
- **Powder_River_Basin_Clinker_USGS:** A (vector) spatial dataset consisting of 29,321 polygon areas or records representing the boundaries of clinker (thermally altered rock caused by the natural burning of coal beds) in the Powder River Basin.
- **Powder_River_Basin_Coalstrip_Coal_Field_Overburden_USGS:** A (vector) spatial dataset consisting of 469 polygon areas or records representing the boundary and overburden (feet) of the Coalstrip Coal Field in the Powder River Basin.
- **Powder_River_Basin_Coalstrip_Coal_Field_Thickness_USGS:** A (vector) spatial dataset consisting of 7 polygon areas or records representing the boundary and thickness (feet) of the Coalstrip Coal Field in the Powder River Basin.
- **Powder_River_Basin_Decker_Coal_Field_Overburden_USGS:** A (vector) spatial dataset consisting of 471 polygon areas or records representing the boundary and overburden (feet) of the Decker Coal Field in the Powder River Basin.
- **Powder_River_Basin_Decker_Coal_Field_Thickness_USGS:** A (vector) spatial dataset consisting of 275 polygon areas or records representing the boundary and thickness (feet) of the Decker Coal Field in the Powder River Basin.
- **Powder_River_Basin_Dietz1_Coal_Bed_Overburden_USGS:** A (raster) spatial dataset representing the boundary and overburden (feet) of the Dietz 1 Coal Bed in the Powder River Basin.
- **Powder_River_Basin_Dietz1_Coal_Bed_Reliability_USGS:** A (raster) spatial dataset representing the reliability of the Dietz 1 Coal Bed measurements, categorized by *measured*, *indicated*, *inferred*, or *hypothetical*.
- **Powder_River_Basin_Dietz1_Coal_Bed_Thickness_USGS:** A (raster) spatial dataset representing the boundary and thickness (feet) of the Dietz 1 Coal Bed in the Powder River Basin.
- **Powder_River_Basin_Dietz2_Coal_Bed_Overburden_USGS:** A (raster) spatial dataset representing the boundary and overburden (feet) of the Dietz 2 Coal Bed in the Powder River Basin.
- **Powder_River_Basin_Dietz2_Coal_Bed_Reliability_USGS:** A (raster) spatial dataset representing the reliability of the Dietz 2 Coal Bed measurements, categorized by *measured*, *indicated*, *inferred*, or *hypothetical*.
- **Powder_River_Basin_Dietz2_Coal_Bed_Thickness_USGS:** A (raster) spatial dataset representing the boundary and thickness (feet) of the Dietz 2 Coal Bed in the Powder River Basin.
- **Powder_River_Basin_Dietz3_Coal_Bed_Overburden_USGS:** A (raster) spatial dataset representing the boundary and overburden (feet) of the Dietz 3 Coal Bed in the Powder River Basin.
- **Powder_River_Basin_Dietz3_Coal_Bed_Reliability_USGS:** A (raster) spatial dataset representing the reliability of the Dietz 3 Coal Bed measurements, categorized by *measured*, *indicated*, *inferred*, or *hypothetical*.
- **Powder_River_Basin_Dietz3_Coal_Bed_Thickness_USGS:** A (raster) spatial dataset representing the boundary and thickness (feet) of the Dietz 3 Coal Bed in the Powder River Basin.
- **Powder_River_Basin_Dietz4_Coal_Bed_Overburden_USGS:** A (raster) spatial dataset representing the boundary and overburden (feet) of the Dietz 4 Coal Bed in the Powder River Basin.
- **Powder_River_Basin_Dietz4_Coal_Bed_Reliability_USGS:** A (raster) spatial dataset representing the reliability of the Dietz 4 Coal Bed measurements, categorized by *measured*, *indicated*, *inferred*, or *hypothetical*.
- **Powder_River_Basin_Dietz4_Coal_Bed_Thickness_USGS:** A (raster) spatial dataset representing the boundary and thickness (feet) of the Dietz 4 Coal Bed in the Powder River Basin.
- **Powder_River_Basin_Felix_Coal_Bed_Overburden_USGS:** A (raster) spatial dataset representing the boundary and overburden (feet) of the Felix Coal Bed in the Powder River Basin.
- **Powder_River_Basin_Felix_Coal_Bed_Reliability_USGS:** A (raster) spatial dataset representing the reliability of the Felix Coal Bed measurements, categorized by *measured*, *indicated*, *inferred*, or *hypothetical*.
- **Powder_River_Basin_Felix_Coal_Bed_Thickness_USGS:** A (raster) spatial dataset representing the boundary and thickness (feet) of the Felix Coal Bed in the Powder River Basin.
- **Powder_River_Basin_Ferry_Coal_Bed_Overburden_USGS:** A (raster) spatial dataset representing the boundary and overburden (feet) of the Ferry Coal Bed in the Powder River Basin.
- **Powder_River_Basin_Ferry_Coal_Bed_Reliability_USGS:** A (raster) spatial dataset representing the reliability of the Ferry Coal Bed measurements, categorized by *measured*, *indicated*, *inferred*, or *hypothetical*.
- **Powder_River_Basin_Ferry_Coal_Bed_Thickness_USGS:** A (raster) spatial dataset representing the boundary and thickness (feet) of the Ferry Coal Bed in the Powder River Basin.
- **Powder_River_Basin_Flowers_Goodale_Deep3_Coal_Bed_Overburden_USGS:** A (raster) spatial dataset representing the boundary and overburden (feet) of the Flowers-Goodale/Deep 3 Coal Bed in the Powder River Basin.
- **Powder_River_Basin_Flowers_Goodale_Deep3_Coal_Bed_Reliability_USGS:** A (raster) spatial dataset representing the reliability of the Flowers-Goodale/Deep 3 Coal Bed measurements, categorized by *measured*, *indicated*, *inferred*, or *hypothetical*.
- **Powder_River_Basin_Flowers_Goodale_Deep3_Coal_Bed_Thickness_USGS:** A (raster) spatial dataset representing the boundary and thickness (feet) of the Flowers-Goodale/Deep 3 Coal Bed in the Powder River Basin.
- **Powder_River_Basin_Gates_Wall_Coal_Bed_Overburden_USGS:** A (raster) spatial dataset representing the boundary and overburden (feet) of the Gates/Wall Coal Bed in the Powder River Basin.
- **Powder_River_Basin_Gates_Wall_Coal_Bed_Reliability_USGS:** A (raster) spatial dataset representing the reliability of the Gates/Wall Coal Bed measurements, categorized by *measured*, *indicated*, *inferred*, or *hypothetical*.
- **Powder_River_Basin_Gates_Wall_Coal_Bed_Thickness_USGS:** A (raster) spatial dataset representing the boundary and thickness (feet) of the Gates/Wall Coal Bed in the Powder River Basin.
- **Powder_River_Basin_Gilette_Coal_Field_Overburden_USGS:** A (vector) spatial dataset consisting of 153 polygon areas or records representing the boundary and overburden (feet) of the Gilette Coal Field in the Powder River Basin.
- **Powder_River_Basin_Gilette_Coal_Field_Thickness_USGS:** A (vector) spatial dataset consisting of 347 polygon areas or records representing the boundary and thickness (feet) of the Gilette Coal Field in the Powder River Basin.
- **Powder_River_Basin_Healy_Lower_Ulm_Coal_Bed_Overburden_USGS:** A (raster) spatial dataset representing the boundary and overburden (feet) of the Healy/Lower Ulm Coal Bed in the Powder River Basin.
- **Powder_River_Basin_Healy_Lower_Ulm_Coal_Bed_Reliability_USGS:** A (raster) spatial dataset representing the reliability of the Healy/Lower Ulm Coal Bed measurements, categorized by *measured*, *indicated*, *inferred*, or *hypothetical*.
- **Powder_River_Basin_Healy_Lower_Ulm_Coal_Bed_Thickness_USGS:** A (raster) spatial dataset representing the boundary and thickness (feet) of the Healy/Lower Ulm Coal Bed in the Powder River Basin.
- **Powder_River_Basin_Lower_Anderson_Coal_Bed_Overburden_USGS:** A (raster) spatial dataset representing the boundary and overburden (feet) of the Lower Anderson Coal Bed in the Powder River Basin.
- **Powder_River_Basin_Lower_Anderson_Coal_Bed_Reliability_USGS:** A (raster) spatial dataset representing the reliability of the Lower Anderson Coal Bed measurements, categorized by *measured*, *indicated*, *inferred*, or *hypothetical*.
- **Powder_River_Basin_Lower_Anderson_Coal_Bed_Thickness_USGS:** A (raster) spatial dataset representing the boundary and thickness (feet) of the Lower Anderson Coal Bed in the Powder River Basin.
- **Powder_River_Basin_Lower_Canyon_Coal_Bed_Overburden_USGS:** A (raster) spatial dataset representing the boundary and overburden (feet) of the Lower Canyon Coal Bed in the Powder River Basin.
- **Powder_River_Basin_Lower_Canyon_Coal_Bed_Reliability_USGS:** A (raster) spatial dataset representing the reliability of the Lower Canyon Coal Bed measurements, categorized by *measured*, *indicated*, *inferred*, or *hypothetical*.
- **Powder_River_Basin_Lower_Canyon_Coal_Bed_Thickness_USGS:** A (raster) spatial dataset representing the boundary and thickness (feet) of the Lower Canyon Coal Bed in the Powder River Basin.
- **Powder_River_Basin_Lower_Felix_Coal_Bed_Overburden_USGS:** A (raster) spatial dataset representing the boundary and overburden (feet) of the Lower Felix Coal Bed in the Powder River Basin.
- **Powder_River_Basin_Lower_Felix_Coal_Bed_Reliability_USGS:** A (raster) spatial dataset representing the reliability of the Lower Felix Coal Bed measurements, categorized by *measured*, *indicated*, *inferred*, or *hypothetical*.
- **Powder_River_Basin_Lower_Felix_Coal_Bed_Thickness_USGS:** A (raster) spatial dataset representing the boundary and thickness (feet) of the Lower Felix Coal Bed in the Powder River Basin.
- **Powder_River_Basin_Lower_Stag_Coal_Bed_Overburden_USGS:** A (raster) spatial dataset representing the boundary and overburden (feet) of the Lower Stag Coal Bed in the Powder River Basin.
- **Powder_River_Basin_Lower_Stag_Coal_Bed_Reliability_USGS:** A (raster) spatial dataset representing the reliability of the Lower Stag Coal Bed measurements, categorized by *measured*, *indicated*, *inferred*, or *hypothetical*.
- **Powder_River_Basin_Lower_Stag_Coal_Bed_Thickness_USGS:** A (raster) spatial dataset representing the boundary and thickness (feet) of the Lower Stag Coal Bed in the Powder River Basin.
- **Powder_River_Basin_McKay_Nance_Deep2_Coal_Bed_Overburden_USGS:** A (raster) spatial dataset representing the boundary and overburden (feet) of the McKay/Nance/Deep 2 Coal Bed in the Powder River Basin.
- **Powder_River_Basin_McKay_Nance_Deep2_Coal_Bed_Reliability_USGS:** A (raster) spatial dataset representing the reliability of the McKay/Nance/Deep 2 Coal Bed measurements, categorized by *measured*, *indicated*, *inferred*, or *hypothetical*.
- **Powder_River_Basin_McKay_Nance_Deep2_Coal_Bed_Thickness_USGS:** A (raster) spatial dataset representing the boundary and thickness (feet) of the McKay/Nance/Deep 2 Coal Bed in the Powder River Basin.
- **Powder_River_Basin_Lower_McKay_S2_Coal_Bed_Overburden_USGS:** A (raster) spatial dataset representing the boundary and overburden (feet) of the Lower McKay/S2 Coal Bed in the Powder River Basin.
- **Powder_River_Basin_Lower_McKay_S2_Coal_Bed_Reliability_USGS:** A (raster) spatial dataset representing the reliability of the Lower McKay/S2 Coal Bed measurements, categorized by *measured*, *indicated*, *inferred*, or *hypothetical*.
- **Powder_River_Basin_Lower_McKay_S2_Coal_Bed_Thickness_USGS:** A (raster) spatial dataset representing the boundary and thickness (feet) of the Lower McKay/S2 Coal Bed in the Powder River Basin.
- **Powder_River_Basin_Murray_Coal_Bed_Overburden_USGS:** A (raster) spatial dataset representing the boundary and overburden (feet) of the Murray Coal Bed in the Powder River Basin.
- **Powder_River_Basin_Murray_Coal_Bed_Reliability_USGS:** A (raster) spatial dataset representing the reliability of the Murray Coal Bed measurements, categorized by *measured*, *indicated*, *inferred*, or *hypothetical*.
- **Powder_River_Basin_Murray_Coal_Bed_Thickness_USGS:** A (raster) spatial dataset representing the boundary and thickness (feet) of the Murray Coal Bed in the Powder River Basin.
- **Powder_River_Basin_Odell_Coal_Bed_Overburden_USGS:** A (raster) spatial dataset representing the boundary and overburden (feet) of the Odell Coal Bed in the Powder River Basin.
- **Powder_River_Basin_Odell_Coal_Bed_Reliability_USGS:** A (raster) spatial dataset representing the reliability of the Odell Coal Bed measurements, categorized by *measured*, *indicated*, *inferred*, or *hypothetical*.
- **Powder_River_Basin_Odell_Coal_Bed_Thickness_USGS:** A (raster) spatial dataset representing the boundary and thickness (feet) of the Odell Coal Bed in the Powder River Basin.
- **Powder_River_Basin_Otter_Coal_Bed_Overburden_USGS:** A (raster) spatial dataset representing the boundary and overburden (feet) of the Otter Coal Bed in the Powder River Basin.
- **Powder_River_Basin_Otter_Coal_Bed_Reliability_USGS:** A (raster) spatial dataset representing the reliability of the Otter Coal Bed measurements, categorized by *measured*, *indicated*, *inferred*, or *hypothetical*.
- **Powder_River_Basin_Otter_Coal_Bed_Thickness_USGS:** A (raster) spatial dataset representing the boundary and thickness (feet) of the Otter Coal Bed in the Powder River Basin.
- **Powder_River_Basin_Pawnee_Coal_Bed_Overburden_USGS:** A (raster) spatial dataset representing the boundary and overburden (feet) of the Pawnee Coal Bed in the Powder River Basin.
- **Powder_River_Basin_Pawnee_Coal_Bed_Reliability_USGS:** A (raster) spatial dataset representing the reliability of the Pawnee Coal Bed measurements, categorized by *measured*, *indicated*, *inferred*, or *hypothetical*.
- **Powder_River_Basin_Pawnee_Coal_Bed_Thickness_USGS:** A (raster) spatial dataset representing the boundary and thickness (feet) of the Pawnee Coal Bed in the Powder River Basin.
- **Powder_River_Basin_Roberts_Terret_Coal_Bed_Overburden_USGS:** A (raster) spatial dataset representing the boundary and overburden (feet) of the Roberts/Terret Coal Bed in the Powder River Basin.
- **Powder_River_Basin_Roberts_Terret_Coal_Bed_Reliability_USGS:** A (raster) spatial dataset representing the reliability of the Roberts/Terret Coal Bed measurements, categorized by *measured*, *indicated*, *inferred*, or *hypothetical*.
- **Powder_River_Basin_Roberts_Terret_Coal_Bed_Thickness_USGS:** A (raster) spatial dataset representing the boundary and thickness (feet) of the Roberts/Terret Coal Bed in the Powder River Basin.
- **Powder_River_Basin_Robinson_Witham_Deep4_Coal_Bed_Overburden_USGS:** A (raster) spatial dataset representing the boundary and overburden (feet) of the Robinson/Witham/Deep 4 Coal Bed in the Powder River Basin.
- **Powder_River_Basin_Robinson_Witham_Deep4_Coal_Bed_Reliability_USGS:** A (raster) spatial dataset representing the reliability of the Robinson/Witham/Deep 4 Coal Bed measurements, categorized by *measured*, *indicated*, *inferred*, or *hypothetical*.
- **Powder_River_Basin_Robinson_Witham_Deep4_Coal_Bed_Thickness_USGS:** A (raster) spatial dataset representing the boundary and thickness (feet) of the Robinson/Witham/Deep 4 Coal Bed in the Powder River Basin.
- **Powder_River_Basin_Roland_Baker_Coal_Bed_Overburden_USGS:** A (raster) spatial dataset representing the boundary and overburden (feet) of the Roland (Baker) Coal Bed in the Powder River Basin.
- **Powder_River_Basin_Roland_Baker_Coal_Bed_Reliability_USGS:** A (raster) spatial dataset representing the reliability of the Roland (Baker) Coal Bed measurements, categorized by *measured*, *indicated*, *inferred*, or *hypothetical*.
- **Powder_River_Basin_Roland_Baker_Coal_Bed_Thickness_USGS:** A (raster) spatial dataset representing the boundary and thickness (feet) of the Roland (Baker) Coal Bed in the Powder River Basin.
- **Powder_River_Basin_Roland_Lower_Rider_Coal_Bed_Overburden_USGS:** A (raster) spatial dataset representing the boundary and overburden (feet) of the Roland Lower Rider Coal Bed in the Powder River Basin.
- **Powder_River_Basin_Roland_Lower_Rider_Coal_Bed_Reliability_USGS:** A (raster) spatial dataset representing the reliability of the Roland Lower Rider Coal Bed measurements, categorized by *measured*, *indicated*, *inferred*, or *hypothetical*.
- **Powder_River_Basin_Roland_Lower_Rider_Coal_Bed_Thickness_USGS:** A (raster) spatial dataset representing the boundary and thickness (feet) of the Roland Lower Rider Coal Bed in the Powder River Basin.
- **Powder_River_Basin_Roland_Taff_Coal_Bed_Overburden_USGS:** A (raster) spatial dataset representing the boundary and overburden (feet) of the Roland (Taff) Coal Bed in the Powder River Basin.
- **Powder_River_Basin_Roland_Taff_Coal_Bed_Reliability_USGS:** A (raster) spatial dataset representing the reliability of the Roland (Taff) Coal Bed measurements, categorized by *measured*, *indicated*, *inferred*, or *hypothetical*.
- **Powder_River_Basin_Roland_Taff_Coal_Bed_Thickness_USGS:** A (raster) spatial dataset representing the boundary and thickness (feet) of the Roland (Taff) Coal Bed in the Powder River Basin.
- **Powder_River_Basin_Roland_Upper_Rider_Coal_Bed_Overburden_USGS:** A (raster) spatial dataset representing the boundary and overburden (feet) of the Roland Upper Rider Coal Bed in the Powder River Basin.
- **Powder_River_Basin_Roland_Upper_Rider_Coal_Bed_Reliability_USGS:** A (raster) spatial dataset representing the reliability of the Roland Upper Rider Coal Bed measurements, categorized by *measured*, *indicated*, *inferred*, or *hypothetical*.
- **Powder_River_Basin_Roland_Upper_Rider_Coal_Bed_Thickness_USGS:** A (raster) spatial dataset representing the boundary and thickness (feet) of the Roland Upper Rider Coal Bed in the Powder River Basin.
- **Powder_River_Basin_Rosebud_Knobloch_Deep1_Coal_Bed_Overburden_USGS:** A (raster) spatial dataset representing the boundary and overburden (feet) of the Rosebud/Knobloch/Deep 1 Coal Bed in the Powder River Basin.
- **Powder_River_Basin_Rosebud_Knobloch_Deep1_Coal_Bed_Reliability_USGS:** A (raster) spatial dataset representing the reliability of the Rosebud/Knobloch/Deep 1 Coal Bed measurements, categorized by *measured*, *indicated*, *inferred*, or *hypothetical*.
- **Powder_River_Basin_Rosebud_Knobloch_Deep1_Coal_Bed_Thickness_USGS:** A (raster) spatial dataset representing the boundary and thickness (feet) of the Rosebud/Knobloch/Deep 1 Coal Bed in the Powder River Basin.
- **Powder_River_Basin_Sheridan_Coal_Field_Overburden_USGS:** A (vector) spatial dataset consisting of 201 polygon areas or records representing the boundary and overburden (feet) of the Sheridan Coal Field in the Powder River Basin.
- **Powder_River_Basin_Sheridan_Coal_Field_Thickness_USGS:** A (vector) spatial dataset consisting of 106 polygon areas or records representing the boundary and thickness (feet) of the Sheridan Coal Field in the Powder River Basin.
- **Powder_River_Basin_Smith_Coal_Bed_Overburden_USGS:** A (raster) spatial dataset representing the boundary and overburden (feet) of the Smith Coal Bed in the Powder River Basin.
- **Powder_River_Basin_Smith_Coal_Bed_Reliability_USGS:** A (raster) spatial dataset representing the reliability of the Smith Coal Bed measurements, categorized by *measured*, *indicated*, *inferred*, or *hypothetical*.
- **Powder_River_Basin_Smith_Coal_Bed_Thickness_USGS:** A (raster) spatial dataset representing the boundary and thickness (feet) of the Smith Coal Bed in the Powder River Basin.
- **Powder_River_Basin_Ucross_Coal_Bed_Overburden_USGS:** A (raster) spatial dataset representing the boundary and overburden (feet) of the Ucross Coal Bed in the Powder River Basin.
- **Powder_River_Basin_Ucross_Coal_Bed_Reliability_USGS:** A (raster) spatial dataset representing the reliability of the Ucross Coal Bed measurements, categorized by *measured*, *indicated*, *inferred*, or *hypothetical*.
- **Powder_River_Basin_Ucross_Coal_Bed_Thickness_USGS:** A (raster) spatial dataset representing the boundary and thickness (feet) of the Ucross Coal Bed in the Powder River Basin.
- **Powder_River_Basin_Upper_Canyon_Cox_Coal_Bed_Overburden_USGS:** A (raster) spatial dataset representing the boundary and overburden (feet) of the Upper Canyon/Cox Coal Bed in the Powder River Basin.
- **Powder_River_Basin_Upper_Canyon_Cox_Coal_Bed_Reliability_USGS:** A (raster) spatial dataset representing the reliability of the Canyon/Cox Coal Bed measurements, categorized by *measured*, *indicated*, *inferred*, or *hypothetical*.
- **Powder_River_Basin_Upper_Canyon_Cox_Coal_Bed_Thickness_USGS:** A (raster) spatial dataset representing the boundary and thickness (feet) of the Canyon/Cox Coal Bed in the Powder River Basin.
- **Powder_River_Basin_Upper_Felix_Coal_Bed_Overburden_USGS:** A (raster) spatial dataset representing the boundary and overburden (feet) of the Upper Felix Coal Bed in the Powder River Basin.
- **Powder_River_Basin_Upper_Felix_Coal_Bed_Reliability_USGS:** A (raster) spatial dataset representing the reliability of the Upper Felix Coal Bed measurements, categorized by *measured*, *indicated*, *inferred*, or *hypothetical*.
- **Powder_River_Basin_Upper_Felix_Coal_Bed_Thickness_USGS:** A (raster) spatial dataset representing the boundary and thickness (feet) of the Upper Felix Coal Bed in the Powder River Basin.
- **Powder_River_Basin_Upper_Ferry_Coal_Bed_Overburden_USGS:** A (raster) spatial dataset representing the boundary and overburden (feet) of the Upper Ferry Coal Bed in the Powder River Basin.
- **Powder_River_Basin_Upper_Ferry_Coal_Bed_Reliability_USGS:** A (raster) spatial dataset representing the reliability of the Upper Ferry Coal Bed measurements, categorized by *measured*, *indicated*, *inferred*, or *hypothetical*.
- **Powder_River_Basin_Upper_Ferry_Coal_Bed_Thickness_USGS:** A (raster) spatial dataset representing the boundary and thickness (feet) of the Upper Ferry Coal Bed in the Powder River Basin.
- **Powder_River_Basin_Upper_Healy_Coal_Bed_Overburden_USGS:** A (raster) spatial dataset representing the boundary and overburden (feet) of the Upper Healy Coal Bed in the Powder River Basin.
- **Powder_River_Basin_Upper_Healy_Coal_Bed_Reliability_USGS:** A (raster) spatial dataset representing the reliability of the Upper Healy Coal Bed measurements, categorized by *measured*, *indicated*, *inferred*, or *hypothetical*.
- **Powder_River_Basin_Upper_Healy_Coal_Bed_Thickness_USGS:** A (raster) spatial dataset representing the boundary and thickness (feet) of the Upper Healy Coal Bed in the Powder River Basin.
- **Powder_River_Basin_Upper_Otter_Coal_Bed_Overburden_USGS:** A (raster) spatial dataset representing the boundary and overburden (feet) of the Upper Otter Coal Bed in the Powder River Basin.
- **Powder_River_Basin_Upper_Otter_Coal_Bed_Reliability_USGS:** A (raster) spatial dataset representing the reliability of the Upper Otter Coal Bed measurements, categorized by *measured*, *indicated*, *inferred*, or *hypothetical*.
- **Powder_River_Basin_Upper_Otter_Coal_Bed_Thickness_USGS:** A (raster) spatial dataset representing the boundary and thickness (feet) of the Upper Otter Coal Bed in the Powder River Basin.
- **Powder_River_Basin_Upper_Rosebud_S1_Coal_Bed_Overburden_USGS:** A (raster) spatial dataset representing the boundary and overburden (feet) of the Upper Rosebud/S1 Coal Bed in the Powder River Basin.
- **Powder_River_Basin_Upper_Rosebud_S1_Coal_Bed_Reliability_USGS:** A (raster) spatial dataset representing the reliability of the Upper Rosebud/S1 Coal Bed measurements, categorized by *measured*, *indicated*, *inferred*, or *hypothetical*.
- **Powder_River_Basin_Upper_Rosebud_S1_Coal_Bed_Thickness_USGS:** A (raster) spatial dataset representing the boundary and thickness (feet) of the Upper Rosebud/S1 Coal Bed in the Powder River Basin.
- **Powder_River_Basin_Upper_Smith_Coal_Bed_Overburden_USGS:** A (raster) spatial dataset representing the boundary and overburden (feet) of the Upper Smith Coal Bed in the Powder River Basin.
- **Powder_River_Basin_Upper_Smith_Coal_Bed_Reliability_USGS:** A (raster) spatial dataset representing the reliability of the Upper Smith Coal Bed measurements, categorized by *measured*, *indicated*, *inferred*, or *hypothetical*.
- **Powder_River_Basin_Upper_Smith_Coal_Bed_Thickness_USGS:** A (raster) spatial dataset representing the boundary and thickness (feet) of the Upper Smith Coal Bed in the Powder River Basin.
- **Powder_River_Basin_Upper_Stag_Coal_Bed_Overburden_USGS:** A (raster) spatial dataset representing the boundary and overburden (feet) of the Upper Stag Coal Bed in the Powder River Basin.
- **Powder_River_Basin_Upper_Stag_Coal_Bed_Reliability_USGS:** A (raster) spatial dataset representing the reliability of the Upper Stag Coal Bed measurements, categorized by *measured*, *indicated*, *inferred*, or *hypothetical*.
- **Powder_River_Basin_Upper_Stag_Coal_Bed_Thickness_USGS:** A (raster) spatial dataset representing the boundary and thickness (feet) of the Upper Stag Coal Bed in the Powder River Basin.
- **Powder_River_Basin_Upper_Witham_Coal_Bed_Overburden_USGS:** A (raster) spatial dataset representing the boundary and overburden (feet) of the Upper Witham Coal Bed in the Powder River Basin.
- **Powder_River_Basin_Upper_Witham_Coal_Bed_Reliability_USGS:** A (raster) spatial dataset representing the reliability of the Upper Witham Coal Bed measurements, categorized by *measured*, *indicated*, *inferred*, or *hypothetical*.
- **Powder_River_Basin_Upper_Witham_Coal_Bed_Thickness_USGS:** A (raster) spatial dataset representing the boundary and thickness (feet) of the Upper Witham Coal Bed in the Powder River Basin.
- **Powder_River_Basin_Werner_Cook_Coal_Bed_Overburden_USGS:** A (raster) spatial dataset representing the boundary and overburden (feet) of the Werner/Cook Coal Bed in the Powder River Basin.
- **Powder_River_Basin_Werner_Cook_Coal_Bed_Reliability_USGS:** A (raster) spatial dataset representing the reliability of the Werner/Cook Coal Bed measurements, categorized by *measured*, *indicated*, *inferred*, or *hypothetical*.
- **Powder_River_Basin_Werner_Cook_Coal_Bed_Thickness_USGS:** A (raster) spatial dataset representing the boundary and thickness (feet) of the Werner/Cook Coal Bed in the Powder River Basin.
- **Powder_River_Basin_Wyodak_Anderson_Coal_Zone_Overburden_USGS:** A (vector) spatial dataset consisting of 2,077 polygon areas or records representing the boundary and overburden (feet) of the Wyodak-Anderson Coal Zone in the Powder River Basin.
- **Powder_River_Basin_Wyodak_Anderson_Coal_Zone_Thickness_USGS:** A (vector) spatial dataset consisting of 1,242 polygon areas or records representing the boundary and thickness (feet) of the Wyodak-Anderson Coal Zone in the Powder River Basin.
- **Williston_Basin_Beulah_Zap_Coal_Zone_Overburden_USGS:** A (vector) spatial dataset consisting of 190 polygon areas or records representing the boundary and overburden (feet) of the Beulah-Zap Coal Zone in the Williston Basin.
- **Williston_Basin_Beulah_Zap_Coal_Zone_Thickness_USGS:** A (vector) spatial dataset consisting of 380 polygon areas or records representing the boundary and thickness (feet) of the Beulah-Zap Coal Zone in the Williston Basin.
- **Williston_Basin_Hagel_Coal_Zone_Overburden_USGS:** A (vector) spatial dataset consisting of 160 polygon areas or records representing the boundary and overburden (feet) of the Hagel Coal Zone in the Williston Basin.
- **Williston_Basin_Hagel_Coal_Zone_Thickness_USGS:** A (vector) spatial dataset consisting of 329 polygon areas or records representing the boundary and thickness (feet) of the Hagel Coal Zone in the Williston Basin.
- **Williston_Basin_Hansen_Coal_Zone_Overburden_USGS:** A (vector) spatial dataset consisting of 403 polygon areas or records representing the boundary and overburden (feet) of the Hansen Coal Zone in the Williston Basin.
- **Williston_Basin_Hansen_Coal_Zone_Thickness_USGS:** A (vector) spatial dataset consisting of 74 polygon areas or records representing the boundary and thickness (feet) of the Hansen Coal Zone in the Williston Basin.
- **Williston_Basin_Harmon_Coal_Zone_Overburden_USGS:** A (vector) spatial dataset consisting of 88 polygon areas or records representing the boundary and overburden (feet) of the Harmon Coal Zone in the Williston Basin.
- **Williston_Basin_Harmon_Coal_Zone_Thickness_USGS:** A (vector) spatial dataset consisting of 93 polygon areas or records representing the boundary and thickness (feet) of the Harmon Coal Zone in the Williston Basin.

**Infrastructure:**

- **AK_resource_data_file_coal_USGS:** A (vector) spatial dataset consisting of 27 point locations or records representing mines, prospects, and mineral occurrences associated with coal (filtered on geologic description) in Alaska.
- **Coal_contamination_sites_EIP:** A (vector) spatial dataset consisting of 737 point locations or records representing groundwater contamination sites associated with coal-fired power plants.
- **Coal_mines_abandoned_SKYTRUTH:** A (vector) spatial dataset consisting of 48,529 point locations or records representing abandoned coal mine sites.
- **Coal_mines_MSHA:** A table consisting of 35,359 records representing coal mines within the United States. This table was filtered for coal mines from the MSHA mine dataset. The original dataset lists all Coal and Metal/Non-Metal mines under MSHA's jurisdiction since 1/1/1970. It includes such information as the status of each mine (Active, Abandoned, NonProducing, etc.), the current owner and operating company, commodity codes and physical attributes of the mine.
- **IL_Colchester_Mines_Py_ISGS:** A (vector) spatial dataset consisting of 475 polygon areas or records representing the boundaries of coal mines that extract coal from the Colchester coal bed in the state of Illinois.
- **IL_Danville_Mines_Py_ISGS:** A (vector) spatial dataset consisting of 292 polygon areas or records representing the boundaries of coal mines that extract coal from the Danville coal bed in the state of Illinois.
- **IL_DekovDavis_Mines_Py_ISGS:** A (vector) spatial dataset consisting of 60 polygon areas or records representing the boundaries of coal mines that extract coal from the Dekoven/Davis coal bed in the state of Illinois.
- **IL_Herrin_Mines_Py_ISGS:** A (vector) spatial dataset consisting of 3,878 polygon areas or records representing the boundaries of coal mines that extract coal from the Herrin coal bed in the state of Illinois.
- **IL_Mines_Active_Py_ISGS:** A (vector) spatial dataset consisting of 422 polygon areas or records representing the boundaries of active coal mines in the state of Illinois.
- **IL_Mines_All_Pt_ISGS:** A (vector) spatial dataset consisting of 7,204 point locations or records representing the boundaries of all coal mines in the state of Illinois.
- **IL_Mines_All_Py_ISGS:** A (vector) spatial dataset consisting of 8,667 polygon areas or records representing the boundaries of all coal mines in the state of Illinois.
- **IL_Seelyville_Mines_Py_ISGS:** A (vector) spatial dataset consisting of 3 polygon areas or records representing the boundaries of coal mines that extract coal from the Seelyville coal bed in the state of Illinois.
- **IL_Springfield_Mines_Pt_ISGS:** A (vector) spatial dataset consisting of 1,422 point locations or records representing the boundaries of coal mines that extract coal from the Springfield coal bed in the state of Illinois.
- **IL_Springfield_Mines_Py_ISGS:** A (vector) spatial dataset consisting of 2,575 polygon areas or records representing the boundaries of coal mines that extract coal from the Springfield coal bed in the state of Illinois.
- **PA_Coal_mining_operations_PASDA:** A (vector) spatial dataset consisting of 13,405 point locations or records representing facilities associated with coal mining operations in the state of Pennsylvania.
- **TX_Coal_mining_site_areas_TRC:** A (vector) spatial dataset consisting of 369 polygon areas or records representing extent of historical coal mining sites in the state of Texas (pre-1977).
- **TX_Coal_mining_sites_TRC:** A (vector) spatial dataset consisting of 353 point locations or records representing historical coal mining sites in the state of Texas (pre-1977).

**Infrastructure network:**

- **Coal_Delivery_Pathways_2011_2016:** A (vector) spatial dataset consisting of 85,072 (poly)line features or records representing individual monthly and annual deliveries of coal from mines to coal-fired power plants from 2011 through 2016. Each delivery record includes location information for both mine and power plants (coordinates, states), primary/secondary transportation modes, if the delivery is inter- or intra- state, average heat sulfur, ash, and mercury content, monthly, annual, and total delivery quantities, and delivery path length (km; calculated in the North America Lambert Conformal Conic projection). The primary data source (EIA) is filtered for domestic US coal deliveries, with integrated mine locations from the MSHA, and coal source region information from the USGS. Data are provided in the “WGS 1984” datum. Field definitions are provided in Supplementary File 3.
- **Coal_Mine_Deliveries_2011_2016:** A (vector) spatial dataset consisting of 968 point locations or records representing individual coal mines with delivery information from 2011 through 2016 integrated from the EIA. Each record includes identification information (MSHA ID#), location information (coordinates), and delivery quantities (count, by year, total). Additional coal source region information was integrated from the USGS. The dataset currently contains 68 unidentified mines with coordinate information. Data are provided in the “WGS 1984” datum. Field definitions are provided in Supplementary File 3.
- **Coal_Mine_Production_2011_2016:** : A (vector) spatial dataset consisting of 1,720 point locations or records representing individual coal mines from the MSHA with production information from 2011 through 2016 integrated from the EIA. Each record includes identification information (MSHA ID#, name, company), location information (coordinates, states, counties, districts), status (active/abandoned/etc.), and other metadata. Additional coal source region information was integrated from the USGS. The production data (by year, total) was integrated from the EIA. Data are provided in the “WGS 1984” datum. Field definitions are provided in Supplementary File 3.
- **Coal_Source_Regions_Production_Deliveries_2011_2016:** A (vector) spatial dataset consisting of 109 polygon areas or records representing coal source regions within the Unites States. This dataset is modified from the “Coal_fields_USGS” dataset. It includes information on deliveries (count, by year, total) and production (mine count, by year, total) quantities from 2011 through 2016 by coal source region. Data are provided in the “WGS 1984” datum. Field definitions are provided in Supplementary File 3.
- **Power_Plant_ByProductsType_2011_2016:** A (vector) spatial dataset consisting of 504 point locations or records representing individual coal-fired power plants with delivery information by coal source region and type of by-product from 2011 through 2016 integrated from the EIA and USGS. This dataset includes quantities of by-products by type and disposition (by year, total). Type of by-products include ash from coal gasification IGCC (Integrated Gasification Combined Combustion Cycle) units, bottom bed ash from FBC (Fluidized Bed Combustion) units, bottom bed ash from standard boiler units, FGD (Flue Gas Desulfurization) gypsum, fly ash from FBC (Fluidized Bed Combustion) units, fly ash from standard boiler PCD (Particulate Control Device) units, fly ash from units with dry FGD (Flue Gas Desulfurization), other FGD (Flue Gas Desulfurization) products, steam sales, other (specifiy via footnote on Schedule 9; explicit type not included in data), and ash totals. Data are provided in the “WGS 1984” datum. Field definitions are provided in Supplementary File 3.
- **Power_Plant_Consumption_2011_2016:** A (vector) spatial dataset consisting of 636 point locations or records representing individual coal-fired power plants with consumption information from 2011 through 2016 integrated from the EIA. Each record includes identification information (Plant ID#), location information (coordinates, states), consumption quantities (by year and coal rank, total), and average heat content (by year and coal rank, total, range). Data are provided in the “WGS 1984” datum. Field definitions are provided in Supplementary File 3.
- **Power_Plant_Deliveries_and_ByProducts_2011_2016:** A (vector) spatial dataset consisting of 505 point locations or records representing individual coal-fired power plants with delivery and by-product information from 2011 through 2016 integrated from the EIA. Each record includes identification information (Plant ID#), location information (coordinates), delivery quantities for each coal source region (by year, total). Data are provided in the “WGS 1984” datum. Field definitions are provided in Supplementary File 3.
- **Power_Plant_Deliveries_by_Coal_Source_Region_2011_2016:** A (vector) spatial dataset consisting of 504 point locations or records representing individual coal-fired power plants with delivery information by coal source region from 2011 through 2016 integrated from the EIA and USGS. Each record includes identification information (Plant ID#), location information (coordinate), stockpile quantities (by year and coal rank, total). Data are provided in the “WGS 1984” datum. Field definitions are provided in Supplementary File 3.
- **Power_Plant_Stockpiles_2011_2016:** A (vector) spatial dataset consisting of 616 point locations or records representing individual coal-fired power plants with stockpile information from 2011 through 2016 integrated from the EIA. Each record includes identification information (Plant ID#), location information (coordinates, states), average stockpile quantities (by year and coal rank, total). Data are provided in the “WGS 1984” datum. Field definitions are provided in Supplementary File 3.

**Samples integrated:**

- **Samples_All:** A table consisting of 36,216 records representing geochemical analyses of coal samples integrated from the datasets within the “Samples original” data category. It contains the metadata for the samples, proximate/ultimate analyses, oxide concentrations, macerals (organic components of the coals), trace element concentrations. Additional metadata fields added include the name of the original dataset (Data_orig_name), source organization (Source_org), link to the data source (Orig_url), and the original datum (Datum), if available. Field definitions are provided in Supplementary File 3.
- **Samples_spatial:** A (vector) spatial dataset consisting of 28,560 point locations or records representing geochemical analyses of coal samples created from available and rectified coordinate information from the “Samples_All” dataset. It contains the same information as in the “Samples_All” dataset. Field definitions are provided in Supplementary File 3.

**Samples original:**

- **AK_Coal_samples_AGDB_USGS:** A table consisting of 6,626 records representing geochemical analyses of coal samples in the state of Alaska. These records were filtered explicitly for coal samples from the Alaska Geochemical Database (Granitto et al., 2013) and processed to include data from multiple related tables (see “Experimental Design, Materials and Methods” section).
- **AK_Holitina_Basin_Coal_samples_ADGGS:** A table consisting of 16 records representing geochemical analyses of coal samples from the Holitina Basin in the state of Alaska. These records were filtered explicitly for coal samples from LePain and Kirkham (2015).
- **AK_Jarvis_creek_coal_samples_ADGGS:** A table consisting of 6 records representing geochemical analyses of coal samples from the Jarvis Creek Coal Field in the state of Alaska. These records include coal samples from Harun and Hendricks (2018).
- **AR_Coal_samples_AGS:** A table consisting of 6 records representing geochemical analyses averaged from 258 coal samples from the state of Arkansas. These records were extracted from the Arkansas Geological Survey webpage (<https://www.geology.arkansas.gov/energy/coal-in-arkansas.html>).
- **Coal_Ash_samples_NETL:** A table consisting of 1,028 records representing rare earth element geochemical analyses from coal and ash (post-combustion coal) samples from various locations within the United States (NETL, 2015). These records were processed to include both coal and ash geochemical analyses for the same samples (see “Experimental Design, Materials and Methods” section).
- **Coal_CCP_samples_USGS:** A table consisting of 449 records representing geochemical analyses from feed coal and coal combustion by-product (CCP) samples from five power plants within the United States (Affolter et al., 2011).
- **Coal_samples_NaCQI_USGS:** A table consisting of 1,458 records representing geochemical analyses of coal samples from the National Coal Quality Inventory (Hatch et al., 2006). These records processed to include data from multiple related tables (see “Experimental Design, Materials and Methods” section).
- **Coal_samples_NGDB_USGS:** A table consisting of 2 records representing coal samples from the USGS National Geochemical Survey DataBase (NGDB) of rock samples. These records were filtered explicitly for coal samples and processed to include data from multiple related tables (see “Experimental Design, Materials and Methods” section).
- **Coal_samples_ Energy_Institute_Coal_Bank_PSU:** A table consisting of 1,468 records representing geochemical analyses of coal samples from the Pennsylvania State University (PSU) Energy Institute’s coal bank.
- **COALQUAL_USGS:** A table consisting of 7,658 records representing geochemical analyses of coal samples from the USGS COALQUAL (Coal Quality) database (Palmer et al., 2015). These records processed to include data from multiple related tables (see “Experimental Design, Materials and Methods” section).
- **Fly_Ash_Samples_Taggart:** A table consisting of 171 records representing rare earth element geochemical analyses of coal fly ash samples originally from coal basins in the United States. These records were extracted from Taggart et al. (2016).
- **IL_Coal_quality_samples_ISGS:** A table consisting of 5,642 records representing geochemical analyses of coal samples within the state of Illinois from the Illinois Coal Quality dataset (ISGS).
- **IN_Coal_quality_samples_DB_IGWS:** A (vector) spatial dataset consisting of 3,125 point locations or records representing geochemical analyses of coal samples from the Indiana Coal Quality Database (Drobniak and Mastalerz, 2012 ; IGWS).
- **KY_Coal_quality_samples_KGS:** A table consisting of 3,238 records representing geochemical analyses of coal samples in the state of Kentucky (KGS).
- **OK_Coal_quality_samples_OGS:** A table consisting of 1,562 records representing geochemical analyses of coal samples within the state of Oklahoma (OGS). These records processed to include data from multiple related tables (see “Experimental Design, Materials and Methods” section). **PRB_Trace_elements_Stratigraphy_USGS:** **:** A (vector) spatial dataset consisting of 3,754 point locations or records representing geochemical analyses of coal samples from well data within the Wyodak-Anderson Coal Zone of the Powder River Basin (USGS; F.U.C.A. 1999)
- **WY_Coal_samples_WSGS:** A table consisting of 7 records representing rare earth element geochemical analyses from coal samples within the State of Wyoming (WSGS). These records were extracted from Sutherland and Cola (2016) and filtered explicitly for coal samples.
